# A high‐dimensional cytometry atlas of peripheral blood over the human life span

**DOI:** 10.1111/imcb.12594

**Published:** 2022-11-06

**Authors:** Sedigheh Jalali, Christopher M Harpur, Adam T Piers, Maria Auladell, Louis Perriman, Shuo Li, Kim An, Jeremy Anderson, Stuart P Berzins, Paul V Licciardi, Thomas M Ashhurst, Igor E Konstantinov, Daniel G Pellicci

**Affiliations:** ^1^ Murdoch Children's Research Institute Melbourne VIC Australia; ^2^ Department of Paediatrics University of Melbourne Melbourne VIC Australia; ^3^ Melbourne Centre for Cardiovascular Genomics and Regenerative Medicine Melbourne VIC Australia; ^4^ Department of Microbiology and Immunology, Peter Doherty Institute for Infection and Immunity University of Melbourne Melbourne VIC Australia; ^5^ Global Product Development Consulting for Infectious Diseases Pharmaceutical Product Development (PPD), Part of Thermo Fisher Scientific Bennekom The Netherlands; ^6^ The Fiona Elsey Cancer Research Institute Ballarat VIC Australia; ^7^ Federation University Ballarat VIC Australia; ^8^ Sydney Cytometry Core Research Facility The University of Sydney and Centenary Institute Sydney NSW Australia; ^9^ School of Medical Sciences, Faculty of Medicine and Health The University of Sydney Sydney NSW Australia; ^10^ Cardiothoracic Surgery Royal Children's Hospital Melbourne VIC Australia

**Keywords:** Cell atlas, high‐dimensional cytometry, immune profiling, ontogeny

## Abstract

Age can profoundly affect susceptibility to a broad range of human diseases. Children are more susceptible to some infectious diseases such as diphtheria and pertussis, while in others, such as coronavirus disease 2019 and hepatitis A, they are more protected compared with adults. One explanation is that the composition of the immune system is a major contributing factor to disease susceptibility and severity. While most studies of the human immune system have focused on adults, how the immune system changes after birth remains poorly understood. Here, using high‐dimensional spectral flow cytometry and computational methods for data integration, we analyzed more than 50 populations of immune cells in the peripheral blood, generating an immune cell atlas that defines the healthy human immune system from birth up to 75 years of age. We focused our efforts on children under 18 years old, revealing major changes in immune cell populations after birth and in children of schooling age. Specifically, CD4^+^ T effector memory cells, Vδ2^+^ gamma delta (γδ)T cells, memory B cells, plasmablasts, CD11c^+^ B cells and CD16^+^CD56^bright^ natural killer (NK) cells peaked in children aged 5–9 years old, whereas frequencies of T helper 1, T helper 17, dendritic cells and CD16^+^CD57^+^CD56^dim^ NK cells were highest in older children (10–18 years old). The frequency of mucosal‐associated invariant T cells was low in the first several years of life and highest in adults between 19 and 30 years old. Late adulthood was associated with fewer mucosal‐associated invariant T cells and Vδ2^+^ γδ T cells but with increased frequencies of memory subsets of B cells, CD4^+^ and CD8^+^ T cells and CD57^+^ NK cells. This human immune cell atlas provides a critical resource to understand changes to the immune system during life and provides a reference for investigating the immune system in the context of human disease. This work may also help guide future therapies that target specific populations of immune cells to protect at‐risk populations.

## Graphical abstract

Age can profoundly affect susceptibility to a broad range of human diseases. Here, using high‐dimensional spectral flow cytometry and data integration, we analyzed more than 50 populations of immune cells in the blood, generating an immune cell atlas that defines the healthy human immune system from birth up to 75 years of age.

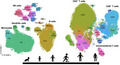

## INTRODUCTION

Most studies that have characterized the human immune system have been carried out in adults and much less is known about how the immune system changes throughout life. Age can greatly influence disease susceptibility, whereby infants and children are more vulnerable than adults for some diseases, while being less susceptible to others.^1^ For example, children are less susceptible to severe acute respiratory syndrome coronavirus 2 (SARS‐CoV‐2) infection, and usually present with mild symptoms or in some cases are asymptomatic, compared with adults.^1^, ^2^, ^3^, ^4^, ^5^, ^6^ Previous studies also reported the same J‐shape curve of severity for other infectious diseases (e.g. SARS‐CoV‐1,^7^ hepatitis A^8^, ^9^ and salmonella).^10^, ^11^ By contrast, respiratory syncytial virus infection,^12^, ^13^, ^14^ diphtheria^15^ and pertussis^16^ exhibit a reverse J‐shaped curve, whereby infants are most susceptible, while adults are largely protected. Moreover, Ebola virus disease,^17^ meningococcal meningitis,^18^ invasive group A streptococcal disease,^19^ pneumococcus^20^ and tuberculosis^21^ exhibited U‐shaped curves of severity throughout life, where infections were more severe in both infants and aging adults (> 45 years old) compared with older children and young adults. Among other pathological conditions, malignancies are more prominent in older individuals, and this has been partially attributed to immune senescence.^22^ Furthermore, the incidence of rejection in ABO‐mismatched heart transplants is greatly reduced in infants if performed less than 14 months of age.^23^, ^24^, ^25^ Thus, the age can greatly impact on disease susceptibility and the composition of the immune system is likely to be an important determining factor for disease incidence and severity.

Traditionally, immune responses are classified as innate and adaptive, both of which play critical roles in the identification and elimination of harmful microorganisms.^26^, ^27^, ^28^, ^29^ The innate immune system consists of natural killer (NK) cells, monocytes, dendritic cells (DCs) and a variety of granulocytes. NK cells can be divided into functionally distinct subsets based on their expression of CD56, CD16 and CD57.^30^, ^31^, ^32^, ^33^ Human monocytes are typically classified as classical, nonclassical and intermediate based on their expression of CD14 and CD16,^34^, ^35^, ^36^ while DCs can be divided into different subsets such as plasmacytoid DCs (pDCs) and myeloid DCs (mDCs) with the expression of cell surface markers HLADR, CD11c and CD123.^36^, ^37^, ^38^

The adaptive immune system is composed of two major populations of lymphocytes, B cells and T cells, which generate humoral immunity and cell‐mediated immune responses, respectively.^26^, ^29^, ^39^ Humoral immunity is predominantly mediated by antibodies that are secreted by B cells, which protects the host from extracellular microorganisms via neutralizing the infectivity of microorganisms, employing phagocytosis to eliminate them and by activating the complement pathway.^26^, ^29^, ^39^ B cells can be divided into naïve and memory subsets, using the markers CD27 and immunoglobulin D (IgD)^40^, ^41^ and these cells are precursors to plasmablasts (CD38^+^CD138^−^) and plasma cells (CD38^+^CD138^+^)^41^, ^42^ and other B‐cell subsets.^41^, ^43^ Cell‐mediated immunity is driven by conventional CD4^+^ T cells and CD8^+^ cytotoxic T cells, which upon activation, release cytokines and cytotoxic granules, respectively. These T cells typically express an alpha‐beta‐T‐cell receptor (αβ‐TCR) that recognize diverse peptide antigens presented by highly polymorphic major histocompatibility complex molecules, which ensures broad protective immunity against a wide variety of pathogens.^27^, ^29^ CD4^+^ T and CD8^+^ T cells can be divided into T naïve, T central memory (T_CM_), T effector memory (T_EM_) and T effector memory CD45RA^+^ (T_EMRA_) cells based on their expression of CCR7 and CD45RA.^44^, ^45^, ^46^, ^47^ CD4^+^ T cells can also be subdivided into T helper (TH) cells, namely TH1, TH2 and TH17 cells, which produce a broad range of different immunoregulatory cytokines that can greatly influence host immunity.^48^, ^49^, ^50^, ^51^, ^52^, ^53^, ^54^ Moreover, another subset of CD4^+^ T cells, termed regulatory T (T_reg_) cells, play a central role by regulating host immune responses.^55^, ^56^

While most studies have focused on major histocompatibility complex/peptide reactive conventional T cells, humans also contain large populations of unconventional T cells that recognize nonpeptide antigens. These cells appear to bridge the gap between innate and adaptive immunity because they recognize antigens via their TCR, but possess qualities of innate cells that allow them to rapidly respond to invading pathogens by secreting cytokines and cytotoxic granules following activation.^57^ For example, mucosal‐associated invariant T (MAIT) cells express a semi‐invariant TCR that recognizes microbial vitamin B derivatives presented by the major histocompatibility complex class I‐like molecule, MR1, while NK T cells express a semi‐invariant TCR that recognizes lipid antigens, and the main population of γδ T cells in humans, Vγ9Vδ2^+^ T cells, express a semi‐invariant TCR that recognizes butyrophilin molecules that sense intracellular phosphoantigens.^57^, ^58^, ^59^

Understanding how the composition of the immune system changes throughout life, will help to identify changes to the immune system caused by disease and may provide important clues on how best to protect at‐risk populations. Here, we combined high‐dimensional spectral flow cytometric immune profiling of peripheral blood with advanced computational methods for data integration and analysis to define more than 50 populations of immune cells to generate an immune cell atlas that defines the healthy human immune system from birth to adults up to 75 years of age. A key focus of this study was the investigation of the immune system in infants and children, which is poorly defined, even though they are at a greater risk for many infectious diseases.

## RESULTS

### Ontogeny of immune cell subsets throughout life

To understand how the immune system changes with age, we analyzed peripheral blood mononuclear cells (PBMCs) of individuals from birth to 75 years of age. Participants up to 18 years old who had undergone minor cardiac corrective surgery were recruited by The Royal Children's Hospital (RCH) and PBMCs from healthy participants over 19 years old were provided by Australian Red Cross. All samples were collected prior to the coronavirus disease 2019 (COVID‐19) outbreak. We divided participants into 11 age groups and each group contained between 6 and 13 donors. Pediatric age groups included participants from day 1 to day 30 after birth (newborns, 0–1 month), from day 31 to day 180 (2–6 months), from day 181 to day 360 (7–12 months), from 13 to 24 months (13–24 months), from 24 months to 4 years (3–4 years), from 4 to 9 years old (5–9 years) and from 9 to 18 years old (10–18 years), whereas adult age groups included 19–30, 31–40, 41–60 and 61–75 years old (Supplementary table 1). PBMCs were analyzed using high‐dimensional spectral flow cytometry, providing information on more than 50 populations of immune cells including conventional T cells, unconventional T cells, B cells, NK cells, monocytes and DCs (Supplementary tables 2–4). Given the womb is considered sterile and infants are exposed to microbial stimuli soon after birth, statistical analyses were performed by comparing the frequency of immune cell populations in newborns with all other age groups.^60^

**Figure 1:**
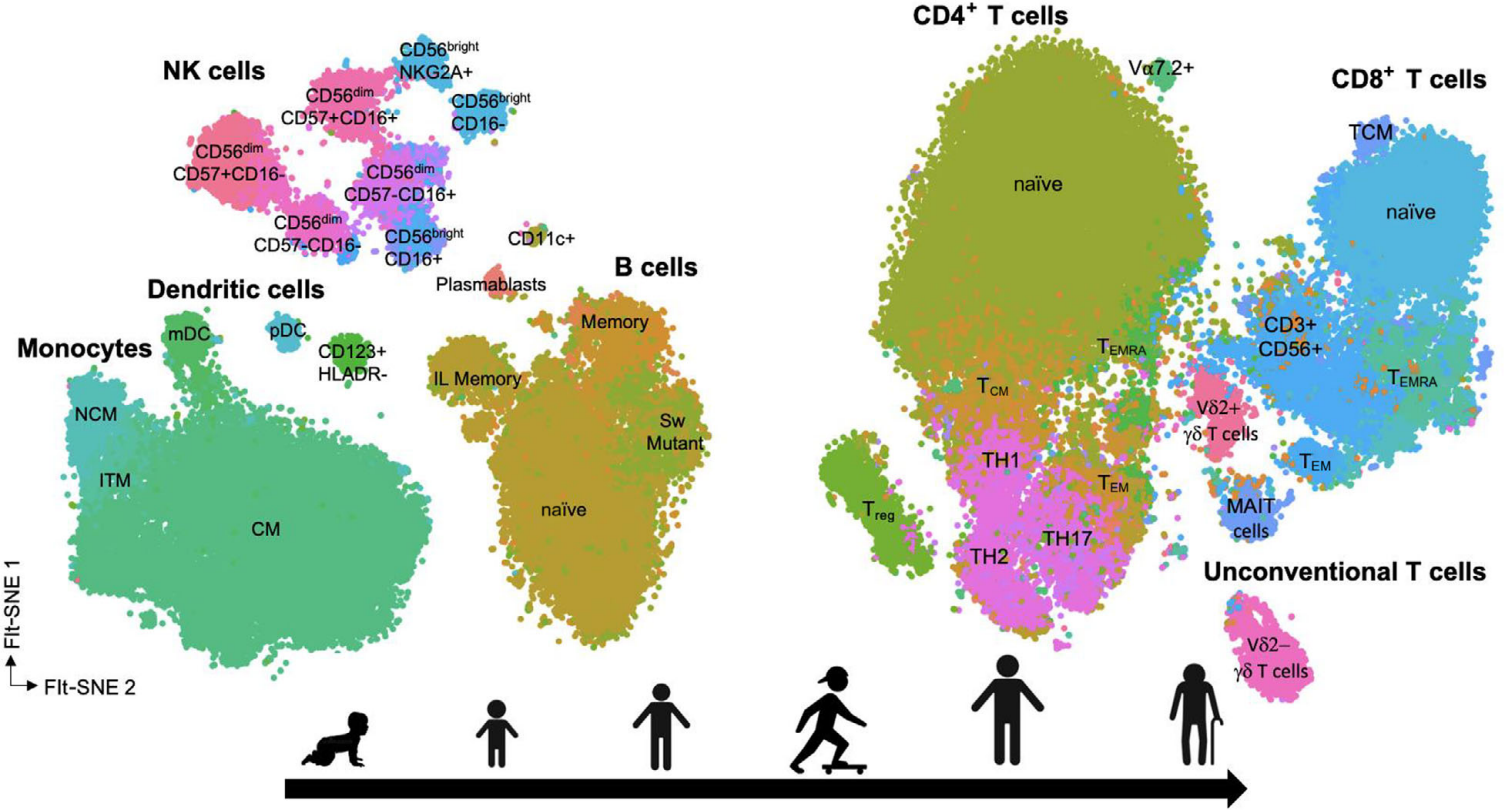
High‐dimensional cytometry atlas of blood immune cell subsets. Dimensionality reduction and visualization method named T‐distributed stochastic neighbor embedding (t‐SNE) and fast Fourier transform–accelerated interpolation‐based t‐SNE (FIt‐SNE) analysis were used to map peripheral blood mononuclear cells (PBMCs) of all participants. CM, classical monocyte; IL, innate‐like; ITM, intermediate monocyte; mDC, myeloid dendritic cell; NCM, nonclassical monocyte; NK, natural killer; pDC, plasmacytoid dendritic cell; T_CM_, T central memory; T_EM_, T effector memory; T_EMRA_, T effector memory CD45RA^+^; TH, T helper; T_reg_, regulatory T.

To map the composition of the immune system throughout life we analyzed data using FlowJo (Supplementary figure 1) and then applied high‐dimensional integration and analysis workflow using the Specter toolkit in R^61^ (Figure 1). In this workflow, we first needed to integrate samples together to minimize technical batch effects, while preserving biological heterogeneity (Supplementary figures 2 and 3). We utilized the reciprocal Principal Component Analysis (rPCA) as implemented in the Seurat toolkit for single‐cell genomics^62^; technical variation as a result of differences in sample preparation, staining or acquisition was corrected, while retaining changes to cellular phenotype that occurred in the different age groups. In brief, data from each batch are projected into each other's PCA space, and pairs linked as mutual nearest neighbors are used to harmonize expression levels. The raw and aligned data were carefully compared and validated to ensure technical effects were removed, but biologically relevant differences were preserved. This approach allowed us to generate a comprehensive map of immune development over the human life span (Figure 1).

### Innate cell subsets alter with age

**Figure 2:**
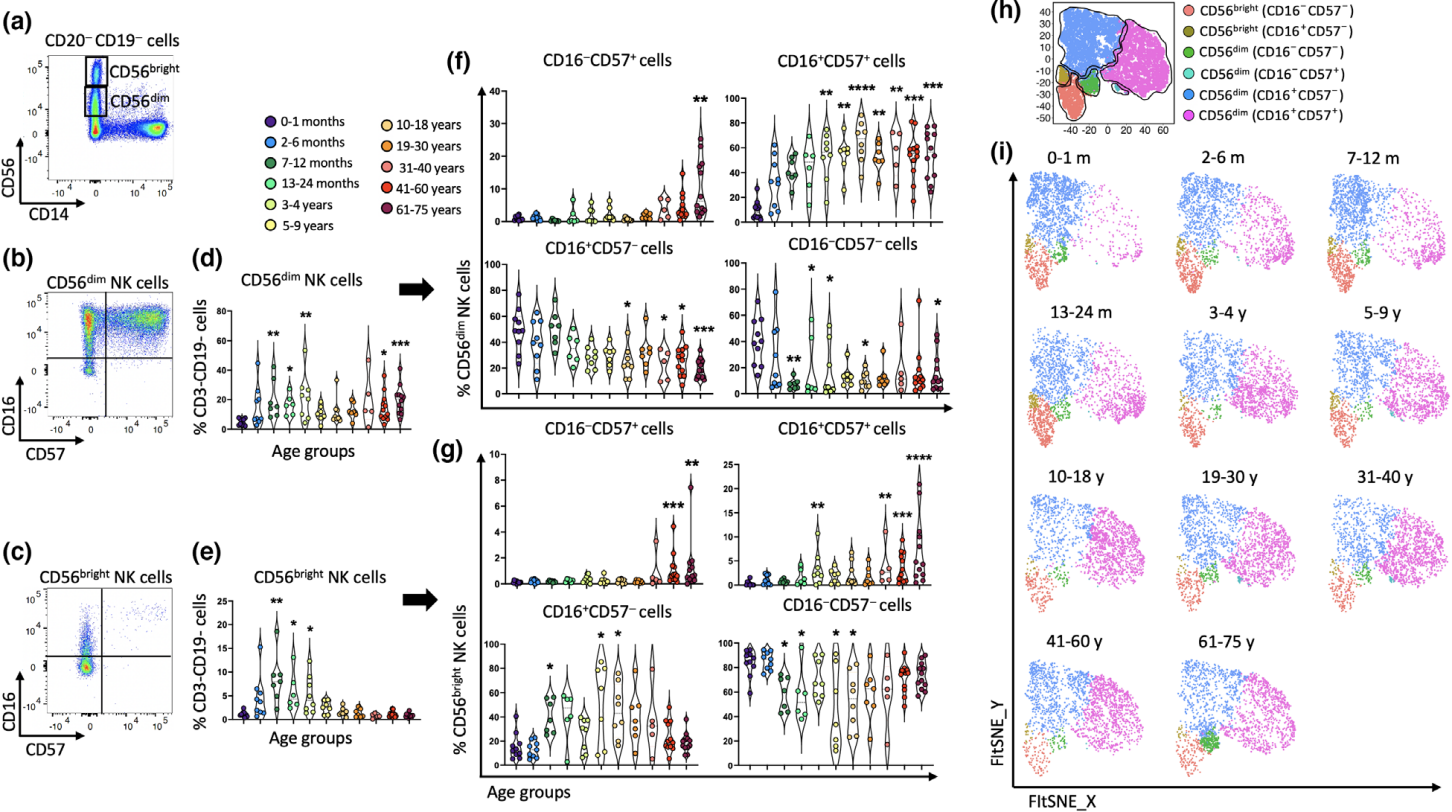
The proportion of different subsets of CD56^dim^ natural killer (NK) cells and CD56^bright^ NK cells changed over time. NK cells of PBMCs from 96 individuals were used for flow cytometric analysis. **(a)** Representative two‐parameter density flow cytometry plots of PBMCs from one of the study participants shows the gating strategy to define CD56^dim^ and CD56^bright^ NK cells. **(b)** CD56^dim^ and **(c)** CD56^bright^ NK cells divided into four subsets based on the expression of CD57 and CD16. Individual‐value violin plots represent the proportion of **(d**) CD56^dim^ and **(e)** CD56^bright^ NK cells. **(f, g)** The violin plots show the frequencies of CD16^+^CD57^+^, CD16^−^CD57^−^, CD16^−^CD57^+^, CD16^+^CD57^−^ CD56^dim^ and CD56^bright^ NK cells among the 11 age groups. Data are shown with median. Each dot represents data from one individual and each color represents one age group. **(h, i)** The transition of different NK cell subsets in the 11 age groups was validated with unsupervised fast Fourier transform–accelerated interpolation‐based T‐distributed stochastic neighbor embedding (FIt‐SNE) analysis. Each color represents a cell subset. The nonparametric Kruskal–Wallis test with Dunn's multiple comparisons test was used for comparing the 0–1‐month age group with all other age groups. *P*‐values are *P* > 0.05 (ns), *P ≤ 0.05; **P ≤ 0.01; ***P ≤ 0.001; ****P ≤ 0.0001.

First, we investigated the proportion of several populations of NK cells (CD56^+^CD14^−^) (Figure 2a–c and Supplementary figure 1) which play critical roles in the first line of defense against disease.^32^, ^63^ Initially, we simply defined NK cells based on the density of CD56 expression into CD56^dim^ and CD56^bright^ cells (Figure 2a) which have distinct functions.^63^, ^64^, ^65^, ^66^, ^67^ The proportion of CD56^dim^ and CD56^bright^ NK cells were the lowest after birth (~5% and 1.04% respectively), and the frequency of CD56^dim^ and CD56^bright^ NK cells significantly increased from 7 to 12 months (~15% and 7.9%, respectively) and remained high up to 4 years of age (~22% and 4.3%, respectively; Figure 2d, e). CD56^dim^ NK cells heavily declined in individuals between 5 and 40 years old, but interestingly increased in individuals above 40 years of age (~20%; Figure 2d), while CD56^bright^ NK cells declined in the 5–9 years age group and represented a minor population thereafter (~2.5–0.8%; Figure 2e). Next, we assessed CD56^dim^ and CD56^bright^ NK cells for the expression of CD16 and CD57 because the functionality and maturation of NK cell subsets are commonly defined by these markers.^63^, ^68^, ^69^ We highlighted four distinct subpopulations of both CD56^dim^ and CD56^bright^ NK cells: CD16^+^CD57^+^, CD16^+^CD57^−^, CD16^−^CD57^+^, CD16^−^CD57^−^ (Figure 2b, c, f, i). CD16^+^CD57^−^CD56^dim^ NK cells were highest in newborns (~48%) and declined with age (~22%), while the proportion of CD16^+^CD57^+^CD56^dim^ NK cells was about 7.8% in the first month after birth, greatly increasing to nearly 32% at 2–6 months of age in infants and to about 55% in adult age groups (Figure 2f, h, i). CD16^−^CD57^+^CD56^dim^ NK cells were low in most age groups (0.1–3.5%) but increased in the oldest age group, representing about 6.2% (Figure 2f). This trend was also seen for CD16^−^CD57^+^CD56^bright^ NK cells which increased in individuals over the age of 40 (Figure 2g). Analysis of CD56^bright^ NK cells revealed that the CD16^−^CD57^−^ subset was highly abundant after birth, although these cells decreased from 7 to 12 months until 18 years of age, replaced by the CD16^+^CD57^−^ subset during this period (Figure 2g, i), while the presence of terminally differentiated CD16^−^CD57^+^ NK cells had a positive correlation with aging (Figure 2f, g). NK cells were assessed for the expression of NKG2A (an inhibitory receptor) and NKG2C (an activating receptor; Supplementary figure 4a–f). Increased expression of NKG2C is thought to correlate with human cytomegalovirus (HCMV) infection,^70^, ^71^, ^72^ and while total NK cells (CD3^−^CD19^−^ cells) increased in early life (2 months to 4 years), the proportion of NKG2C^+^ NK cells did not significantly increase after birth (0–1 month; Supplementary figure 5a, b). The proportion of NKG2A^+^CD56^dim^ NK cells decreased over time, while CD56^bright^ NK cells expressed high levels of NKG2A from infancy until late adulthood (Supplementary figure 4c, e, f). By contrast, NKG2C was mainly expressed by CD56^dim^ NK cells and increased in individuals above 3 years of age (Supplementary figure 4d, f).

We also investigated the proportion of other innate immune cell subsets including classical monocytes (CM) (CD14^+^CD16^−^), intermediate monocytes (ITM) (CD14^+^CD16^+^), nonclassical monocytes (NC) (CD14^−^CD16^+^), myeloid DCs (mDCs) (CD14^−^CD16^−^CD11c^+^) and plasmacytoid DCs (pDCs) (CD14^−^CD16^−^CD11c^−^CD123^+^) as a proportion of CD3^−^CD19^−^ cells (Supplementary figure 6a–f). Classical monocytes were the major subset in all age groups, representing between 30% and 54%, although considerable variability was observed for this population between individuals (Supplementary figure 6c, e, f). The proportion of CM were significantly lower in the 2–6‐month, 7–12‐month and 61–75‐year age groups compared with the 0–1‐month age group (Supplementary figure 6c). ITM and NC cells appeared lower in adults compared with children; however, they were only significantly different in the 31–40‐year age group compared with the 0–1‐month age group for ITM (Supplementary figure 6c). Total DCs increased in the 5–9‐year and 10–18‐year age groups (6.2% and 6.4%, respectively) compared with newborns (~3.7%), and then declined in adults (Supplementary figure 6c, e, f). The proportion of mDCs was highly variable between individuals, representing about 30–50% of total DCs, while the proportion of pDCs was about 12% of total DCs during the first month of life, which increased to nearly 36% in the 13–24‐month age group, before sharply declining about 8% in adults (Supplementary figure 6d–f).

### Age‐related changes to T‐cell subsets

**Figure 3:**
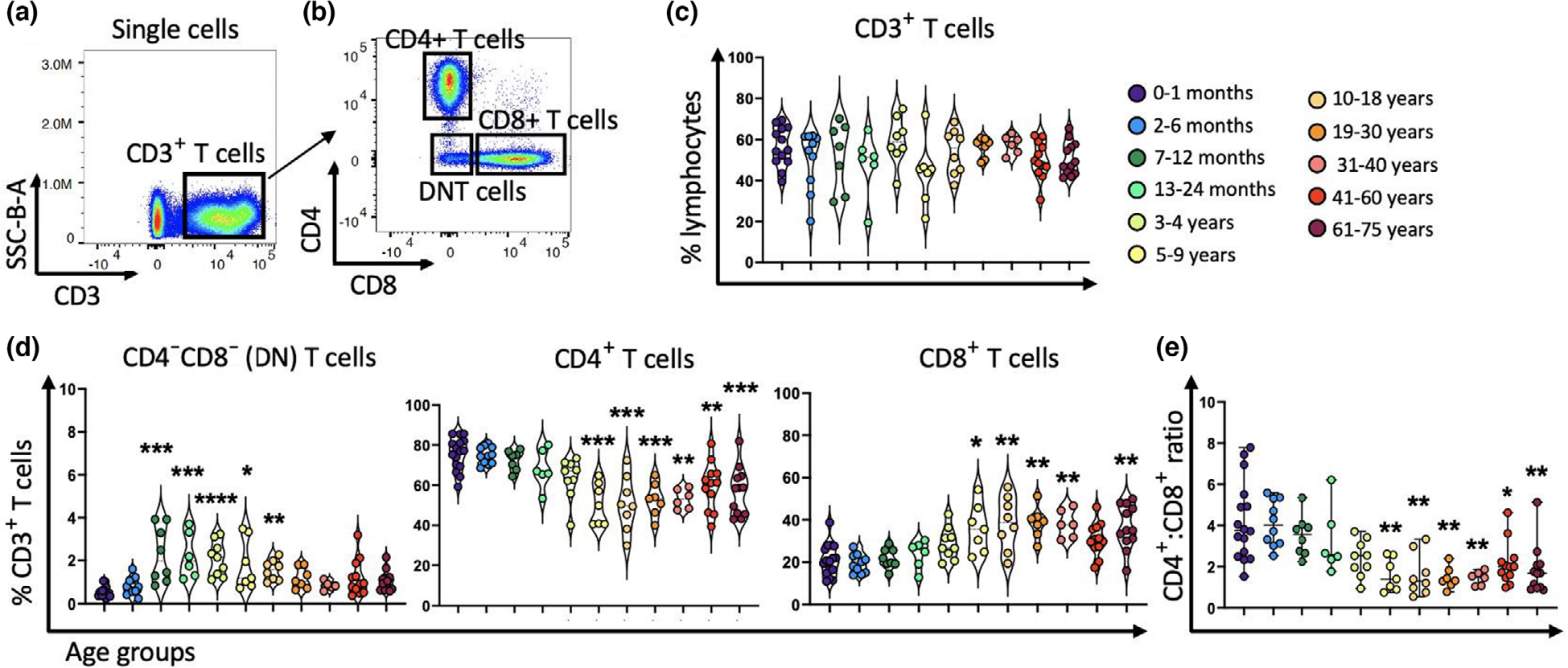
The proportion of total CD3^+^ T cells did not change over time, while aging affects the proportion of CD4^+^ T cells, CD8^+^ T cells and CD4^−^CD8^−^ T cells. Different subsets of T cells from 96 human PBMC samples were used for flow cytometric analysis. Representative two‐parameter density flow cytometry plots of PBMCs from one of the study participants show gating strategy to define **(a)** CD3^+^ T cells, **(b)** CD4^+^ T cells, CD8^+^ T cells and CD4^−^CD8^−^ T cells of PBMCs. **(c, d)** The violin plots represent the proportion of CD3^+^ T cells of live lymphocytes and CD4^+^ T cells, CD8^+^ T cells and CD4^−^CD8^−^ T cells of CD3^+^ T cells among the 11 age groups. Data are shown with median. Each dot represents data from one individual and each color represents one age group. **(e)** The individual‐value plot shows CD4^+^:CD8^+^ T cells ratio throughout life which is shown with median and range. Each dot represents data from one individual and each color represents one age group. The nonparametric Kruskal–Wallis test with Dunn's multiple comparisons test was used for comparing the 0–1‐month age group with all other age groups. *P*‐values are *P* > 0.05 (ns), *P ≤ 0.05; **P ≤ 0.01; ***P ≤ 0.001; ****P ≤ 0.0001. DN, double negative; SSC, side scatter.

Next, we examined the proportion of total CD3^+^ T cells (Supplementary figure 1 and Figure 3a), and CD4^+^, CD8^+^ and double‐negative (DN) T cells (CD4^−^CD8^−^) as a percentage of CD3^+^ T cells (Figure 3b). We found similar frequencies of CD3^+^ T cells in all age groups (~48–56%; Figure 3c). Analysis of CD4^+^ and CD8^+^ T cells revealed higher levels of CD4^+^ T cells than CD8^+^ T cells in all age groups, although the frequency of CD8^+^ T cells significantly increased throughout life commencing from 5 to 9 years of age (~35%) when compared with newborns (~21%; Figure 3d). The ratio of CD4^+^:CD8^+^ T cells was about 4:1 in newborns, almost reaching about 1:1 in the 5–9‐year age group and remaining steady thereafter (Figure 3e). Notably, the proportion of DN T cells (Figure 3b) was low in the first 6 months of life (~0.5–0.8%), increasing afterwards and representing about 2% of total CD3^+^ T cells from 7–12 months to 10–18 years, before decreasing in adults and elderly individuals (~1%; Figure 3d). Thus, age profoundly affected the proportions of CD4^+^, CD8^+^ and DN T cells, with children containing much higher levels of DN T cells than infants and adults.

**Figure 4:**
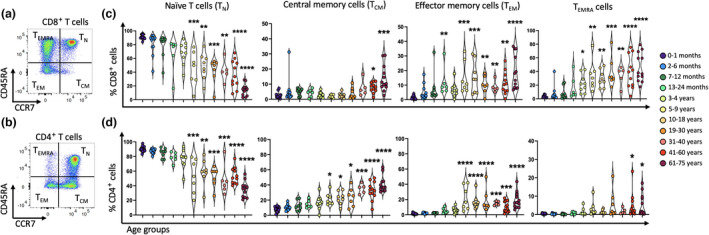
The proportion of CD8^+^ T and CD4^+^ T naïve cells decreased over time and replaced with different subsets of memory cells. CD8^+^ T cells and CD4^+^ T cells of PBMCs from 96 individuals were used for flow cytometric analysis. **(a, b)** Representative two‐parameter density flow cytometry plots of PBMCs from one of the study participants show CD8^+^ naïve T cells and three memory cell subsets as well as CD4^+^ naïve T cells and three memory cell subsets based on the expression of CCR7 and CD45RA. **(c)** The violin plots show the proportion of T naïve (T_N_), T central memory (T_CM_), T effector memory (T_EM_) and T effector memory CD45RA^+^ (T_EMRA_) CD8^+^ T cells among the 11 age groups. **(d)** Individual‐value violin plots represent the proportion of naïve, T_CM_, T_EM_ and T_EMRA_ CD4^+^ T cells among the 11 age groups. Data are shown with median. Each dot represents data from one individual and each color represents one age group. The nonparametric Kruskal–Wallis test with Dunn's multiple comparisons test was used for comparing the 0–1‐month age group with all other age groups. *P*‐values are *P* > 0.05 (ns), *P ≤ 0.05; **P ≤ 0.01; ***P ≤ 0.001; ****P ≤ 0.0001

**Figure 5:**
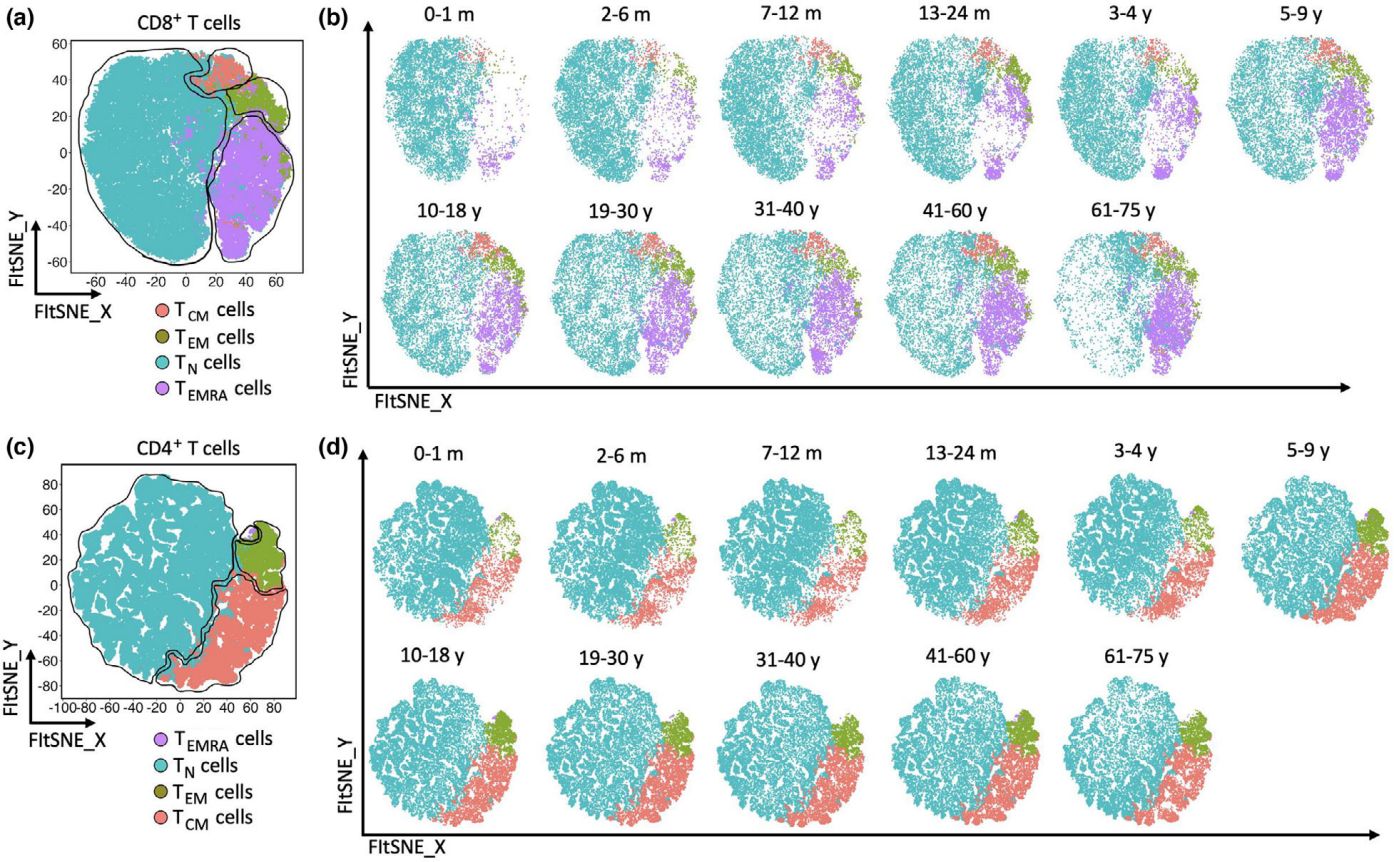
Validation of transition from naïve to memory T‐cell subsets. Fast Fourier transform–accelerated interpolation‐based T‐distributed stochastic neighbor embedding (FIt‐SNE) plots represent the transition from naïve to memory cells of **(a, b)** CD8^+^ T cells and **(c, d)** CD4^+^ T cells in the 11 age groups over time which was validated using unsupervised t‐SNE analysis. Each color represents one T‐cell subset. T_CM_, T central memory; T_EM_, T effector memory; T_EMRA_, T effector memory CD45RA^+^; T_N_, T naïve.

CD4^+^ and CD8^+^ T cells were then assessed for their expression of CCR7 and CD45RA to determine the proportion of naïve *versus* memory T‐cell subsets (Figure 4a, b). The proportion of naïve (CCR7^+^CD45RA^+^) CD4^+^ and CD8^+^ T cells was higher in the younger age groups, while the proportion of T_CM_ (CCR7^+^CD45RA^−^), T_EM_ (CCR7^−^CD45RA^−^) and T_EMRA_ (CCR7^−^CD45RA^+^) cells increased with age (Figure 4c, d). The reduction of naïve T cells was especially pronounced for CD8^+^ T cells, nearly 15% in 61–75‐year‐old individuals, compared with nearly 90% in newborns (Figure 4c). While these results are not unexpected because of increased pathogen exposure throughout life,^45^, ^73^, ^74^ we identified other potentially important changes in these populations. For example, T_CM_ cells were predominant among CD4^+^ memory T cells in adults and continued to increase in frequency throughout life (Figure 4d), while CD8^+^ T_CM_ cells only increased to about 8–10% in individuals after 40 years of age (Figure 4c). Moreover, CD8^+^ T_EMRA_ cells were first detected soon after birth, increasing in an age‐related manner (Figure 4c), while CD4^+^_TEMRA_ cells were barely detectable in children and were only significantly higher in the 41–60‐year and 61–75‐year age groups (2.1% and 1.6%, respectively), compared to newborns (0.6%; Figure 4d). The transition from naïve to memory CD4^+^ and CD8^+^ T cells over time was validated using unsupervised fast Fourier transform–accelerated interpolation‐based T‐distributed stochastic neighbor embedding (FIt‐SNE) analysis (Figure 5a–d). Taken together, these data suggest durable T‐cell activation following ongoing antigen exposure throughout life, evidenced by an age‐related increases of CD4^+^ and CD8^+^ memory T‐cell subsets.

**Figure 6:**
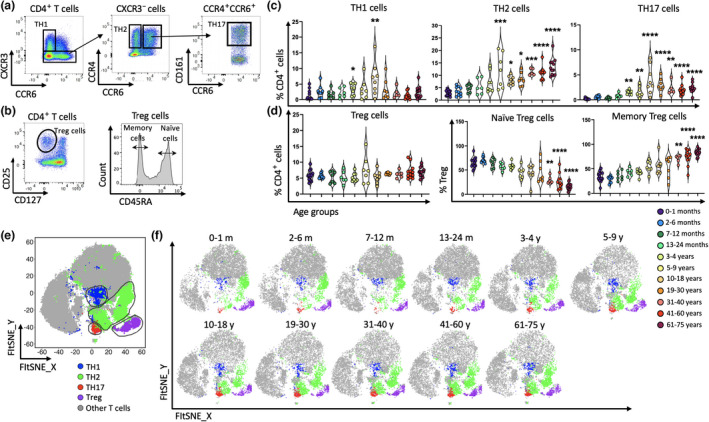
The proportion of regulatory T (T_reg_) cells did not alter with age, while aging significantly affect the proportion of helper T (TH) cell subsets over time. CD4^+^ T cells of PBMCs from 96 individuals were used for further flow cytometric analysis. Representative two‐parameter density flow cytometry plots of PBMCs from one of the study participants show **(a)** TH1, TH2, TH17 and **(b)** total T_reg_, naïve and memory T_reg_ cells. Individual‐value violin plots represent the proportion of **(c)** TH cell subsets and **(d)** Treg cells among the 11 age groups. Data are shown with median. Each dot represents data from one individual and each color represents one age group. **(e, f)** The transition of different CD4^+^ T‐cell subsets in the 11 age groups over time was validated with unsupervised fast Fourier transform–accelerated interpolation‐based T‐distributed stochastic neighbor embedding (FIt‐SNE) analysis. Each color represents a cell subset. The nonparametric Kruskal–Wallis test with Dunn's multiple comparisons test was used for comparing the 0–1‐month age group with all other age groups. *P*‐values are *P* > 0.05 (ns), *P ≤ 0.05; **P ≤ 0.01; ***P ≤ 0.001; ****P ≤ 0.0001.

Next, we investigated the proportion of T_reg_ (CD4^+^CD25^+^CD127^−^) cells and CD4^+^ TH subsets, defined using chemokine receptors^75^ and c‐type lectin, CD161.^76^ These included TH1 (CXCR3^+^CCR6^−^), TH2 (CXCR3^−^CCR4^+^CCR6^−^), TH17 (CXCR3^−^CCR4^+^CCR6^+^CD161^+^) cells and naïve (CD45RA^+^) and memory (CD45RA^−^) T_reg_ cells (Figure 6a, b and Supplementary figure 7). Analysis of the different TH subsets revealed striking differences throughout life (Figure 6c–f), while the overall proportion of T_reg_ cells was similar over the different ages, ranging from 4.7% to 7% of CD4^+^ T cells (Figure 6d–f). TH1 cells increased in the 3–4‐year age group (~3.7%), peaking in children between 10 and 18 years old (~6.4%), before declining in adults (~1.4–2.4%; Figure 6c, e, f). Moreover, the proportion of TH2 cells was low after birth representing about 2.8% of CD4^+^ T cells, which increased to approximately 12% in the 5–9‐year age group and remained high in the 61–75‐year age group (8–13%; Figure 6c, e, f). The proportion of TH17 cells was virtually undetectable in the first year of life (< 0.1%); however, these cells significantly increased in the 3–4‐year age group (~1.5%), peaking in children between 10 and 18 years of age (~3.1%), and remaining high in older age groups (1.6–2.6%; Figure 6c, e, f). Further breakdown of T_reg_ cells into naïve and memory subsets revealed naïve T_reg_ decreased, while memory T_reg_ increased with age (Figure 6d), similar to the age‐related trends observed for naïve and memory CD4^+^ and CD8^+^ T cells (Figures 4 and 5). Thus, while the overall frequency of T_reg_ cells remained stable over time, the different subsets of functionally distinct TH subsets changed considerably, particularly during childhood and in later life.

### Major changes to unconventional T cells in early life

**Figure 7:**
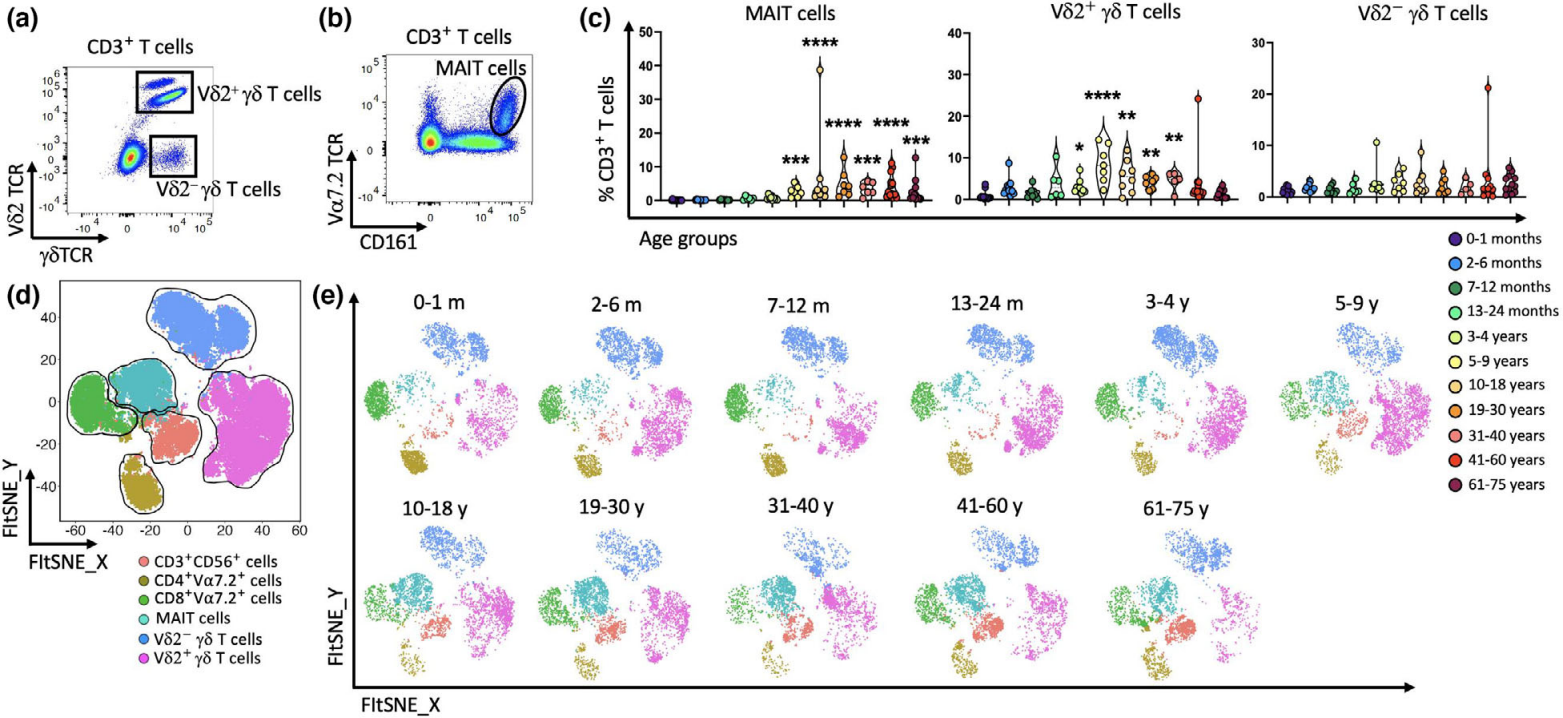
The proportion of unconventional T cells increased in children and young adults following a decline in adults’ older age groups. Unconventional T‐cell subsets of PBMCs from 96 individuals were used for flow cytometric analysis. **(a, b)** Representative two‐parameter density flow cytometry plots of PBMCs from one of the study participants show gating strategy to define two γδ T‐cell subsets and mucosal‐associated invariant T (MAIT) cells. **(c)** Individual‐value violin plots represent the proportion of Vδ2^+^ and Vδ2^−^ γδ T cells, and MAIT cells among the 11 age groups. Data are shown with median. Each dot represents data from one individual and each color represents one age group. **(d, e)** The transition of unconventional T cells over time among the 11 age groups was validated using unsupervised T‐distributed stochastic neighbor embedding (t‐SNE) analysis. Each color represents one cell subset. The nonparametric Kruskal–Wallis test with Dunn's multiple comparisons test was used comparing the 0–1‐month age group with all other age groups. *P*‐values are *P* > 0.05 (ns), *P ≤ 0.05; **P ≤ 0.01; ***P ≤ 0.001; ****P ≤ 0.0001.

To study the changes to unconventional T cells over time, we used Vα7.2^+^CD161^+^ to define MAIT cells and divided γδ T cells into two subsets based on the expression of Vδ2 (Figure 7a, b and Supplementary figure 8). Remarkably, MAIT cells were virtually absent in the first few years of life (< 0.08%), significantly increasing to about 2.3% in the 5–9‐year age group, reaching nearly 4.3% in adults aged 19–30 years and trending lower in individuals above the age of 60 (~0.9%; Figure 7c–e). Similarly, the proportion of Vδ2^+^ γδ T cells was low after birth (< 0.4%), significantly increasing in individuals above 3–4 years of age (~2.1%), peaking in the 5–9‐year age group (~8.1%), before decreasing in adults above 40 years old (~1.8%; Figure 7c–e). The proportion of Vδ2^−^ γδ T cells was typically low in peripheral blood (~0.9–2.7%) and no significant change was observed over time (Figure 7c–e). Like for NK cells, CD56 can be expressed by subsets of T cells (e.g. CD8^+^ T cells and NKT cells).^77^, ^78^ Our results showed that the frequency of CD3^+^CD56^+^ T cells increased over time (Figure 7d, e). We also observed that populations of CD161^−^ Vα7.2^+^CD4^+^ T cells and CD161^−^Vα7.2^+^CD8^+^ T cells were prominent in early life and tended to decrease over time (Figure 7d, e). These likely comprise mostly conventional T cells that happen to express Vα7.2 but could include CD1b‐restricted germline‐encoded mycolyl lipid–reactive (GEM) T cells,^79^ although further studies are required to validate the frequency of CD1‐restricted T cells throughout life. Nonetheless, given that unconventional T cells play an important role in antimicrobial immunity,^80^, ^81^, ^82^ the near absence of MAIT cells and Vδ2^+^ γδ T cells in early life and then the reduction of these cells in older individuals might affect the immune response to a broad range of infectious diseases.^83^, ^84^

### Memory B cells accumulate with age

**Figure 8:**
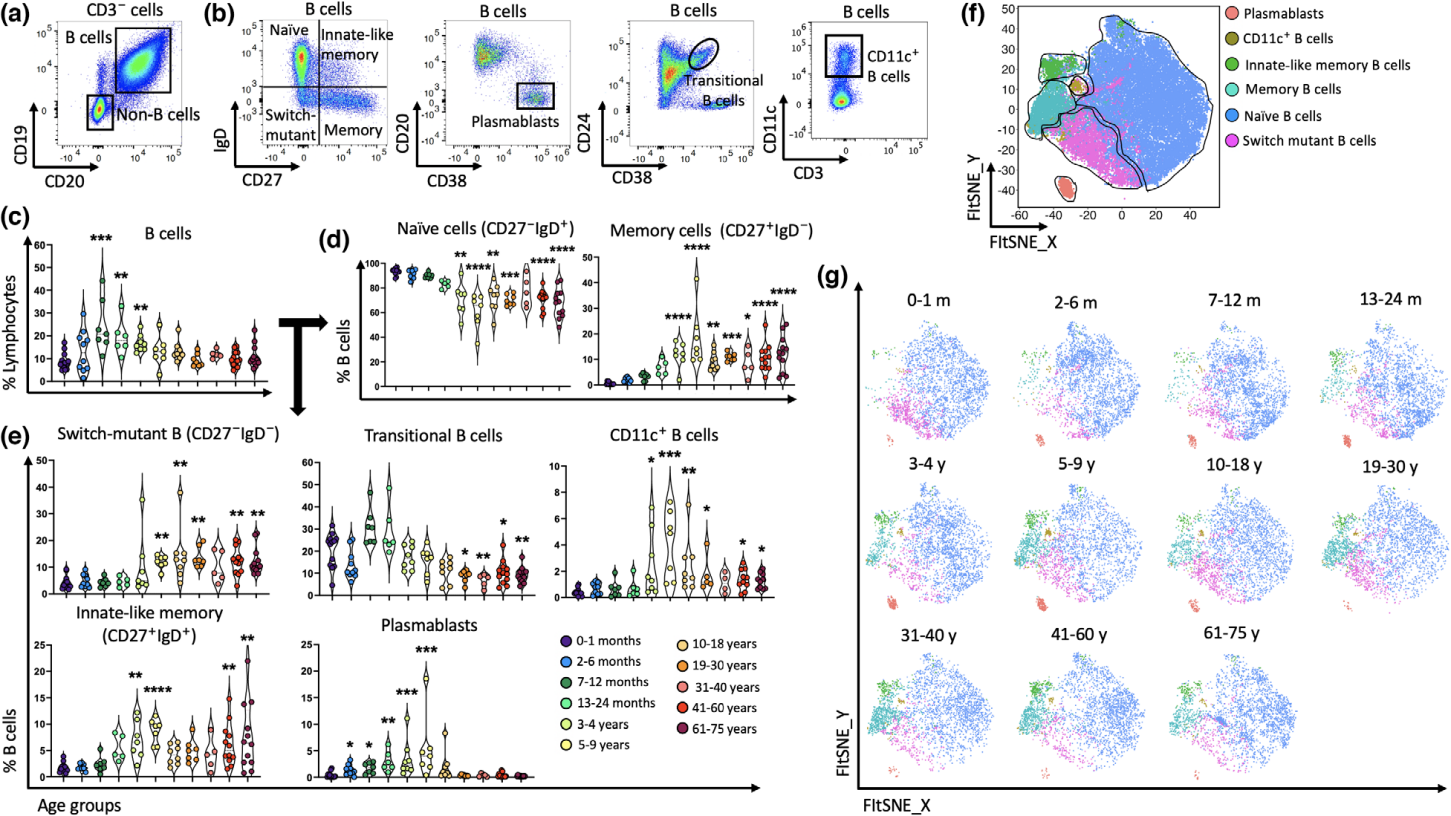
Aging alters the proportion of different subsets of B cells. B cells of PBMCs from 96 individuals were used for flow cytometric analysis. **(a, b)** Representative two‐parameter density flow cytometry plots of PBMCs from one of the study participants show gating strategy to define total B cells and seven subsets of B cells. **(c–e)** The violin plots show the proportion of total B cells, naïve B cells, memory B cells, switch mutant B cells, transitional B cells, CD11c^+^ B cells, innate‐like memory cells and plasmablasts among the 11 age groups. Data are shown with median. Each dot represents data from one individual and each color represents one age group. **(f, g)** FIt‐NSE plots represent the transition from naïve to memory B cells and other B‐cell subsets over time which was validated using unsupervised T‐distributed stochastic neighbor embedding (t‐SNE) analysis. Each color represents a cell subset. The nonparametric Kruskal–Wallis test with Dunn's multiple comparisons test was used for comparing the 0–1‐month age group with all other age groups. *P*‐values are *P* > 0.05 (ns), *P ≤ 0.05; **P ≤ 0.01; ***P ≤ 0.001; ****P ≤ 0.0001.

Total B cells (and subsets thereof) were defined as outlined in Supplementary figure 1 and Figure 8a, b. The proportion of total B cells (CD19^+^CD3^−^) was nearly 7% in newborns, significantly increasing to 20% in 7–12‐month‐old participants. These cells remained high to up until 4 years old (~16%), before gradually declining to about 8–13% after 5 years of age (Figure 8c). Dividing B cells into memory and naïve subsets using CD27 and IgD (Figure 8b) revealed high levels of CD27^−^IgD^+^ naïve B cells throughout life, typically representing 66–94% of total B cells. These cells decreased in frequency and were statistically lower in all age groups over 3 years of age when compared with newborns (Figure 8d). Conversely, CD27^+^IgD^−^ memory B cells were low in early life (~0.3%) and increased from 3 years of age (~12%; Figure 8d), and a similar trend was observed for CD27^+^IgD^+^ innate‐like (IL) memory B cells. CD27^−^IgD^−^‐switched mutant (exhausted) B cells were about 3% during the first 4 years of life, increasing to approximately 12% in the 5–9‐year age group and remaining high over time (Figure 8e–g). Plasmablasts (CD20^−^CD38^+^; Figure 8b) were low in proportion after birth (< 0.1), increased in the 2–6‐month age group (~1%), peaking in the 5–9‐year age group (~4%), before rapidly declining in adults (~0.9–0.2%), while transitional B cells made up between 13% and 30% of B cells in infants and children and were significantly lower in all adult age groups (8–9%; Figure 8e).

Furthermore, CD11c^+^ B cells, which are thought to have a role in autoimmune responses,^43^ exhibited a similar trend to other memory B cells, increasing in children aged 3–4 years (~1.4%), peaking in children 5–9 years (~4.8%) and decreasing in adults (~1%) compared with newborns (< 0.3%; Figure 8e). The transition from naïve to memory B cells over time was validated using unsupervised FIt‐SNE analysis (Figure 8f, g). The notable increase of memory B cells and plasmablasts in early life paralleled the changes observed for CD4^+^ T_EM_ cells (Figure 5), suggesting coordinated T‐cell– and B‐cell–mediated immunity in early childhood years.

Apart from increases in memory T‐ and B‐cell populations during ontogeny (Figures 4 and 8), other trends also emerged. MAIT cells, Vδ2^+^ γδ T cells and plasmablasts all increased from birth to 5–9 years old before declining in children 10–18 years old, while MAIT cells and Vδ2^+^ γδ T cells continued to decrease in aging adults (Supplementary figure 9a, b).^85^, ^86^ Moreover, B cells and CD56^bright^ NK cells trended similarly by peaking at about 7–12 months of age before declining as children reached adulthood (Supplementary figure 9c).

The number of individuals in each group limited immune profiling based on gender, although several age groups contained enough numbers for comparison, including those within the 2–6‐month, 7–12‐month, 5–9‐year, 19–30‐year and 41–60‐year age groups. Notably, there were no significant differences in these age groups for most immune cell subsets, except for those aged between 41 and 60 years old, which showed higher levels of naïve CD8^+^ T cells and lower levels of CD8^+^ T_EMRA_ cells, CD4^+^ T_EMRA_ cells, transitional B cells and myeloid DCs in males compared with females (Supplementary figure 10a–e).

## DISCUSSION

Both the innate and adaptive arms of the immune system are affected by age, and these likely influence disease susceptibility and severity. Using high‐dimensional spectral flow cytometry, we provide a human immune cell atlas of peripheral blood from birth up to 75 years of age. Our study was focused on children below the age of 18 as there is paucity of data from infants and children. Many changes to the immune system occur in the first several years of life, with the immune system in newborns presenting as immature or underdeveloped, with little evidence of immunological memory. We demonstrated that the application of novel data integration and analysis approaches typically used for single‐cell RNA sequencing (sc‐RNAseq) data^62^ was also beneficial for large‐scale analysis of flow cytometry data. Together, these technologies have allowed us to survey the immune landscape throughout life, highlighting key changes to the composition of the immune system after birth, in school‐age children and in older adults.

We observed pronounced heterogeneity not only in T‐cell populations but also within B cells and NK cells, which aligns with previous studies that showed age‐related changes among some of these cell types when analyzed using ‐sc‐RNAseq.^68^, ^87^, ^88^ Our work covers a greater range of age groups, providing novel insight into the immune system of infants and children. For example, analysis of innate cells in this study revealed greater variation in children compared with the adults. Specifically, cytotoxic CD56^dim^ and cytokine‐producing CD56^bright^ NK cells^63^, ^64^, ^65^ upregulate CD57 to become terminally differentiated cells,^89^ with the lowest proportion of CD57^+^ cells found in newborns. Moreover, the expression of NKG2C, which is thought to correlate with human cytomegalovirus (HCMV) infection,^71^, ^90^ appeared higher on CD56^dim^ NK cells compared with CD56^bright^ NK cells between the ages of 3 and 18 years. The observed differences in NK cell subsets, particularly in children, suggest that these cells play an important role in the first line of defense against infectious pathogens. Among the B‐cell subsets analyzed, we provide more detailed information about CD11c^+^ B cells and plasmablasts. While the proportion of CD11c^+^ B cells has been previously reported to be low in healthy adults,^41^, ^43^, ^91^ we revealed that the frequency of these cells among children between 3 and 18 years was much higher than adults. This B‐cell subset is increased in autoimmune diseases such as systemic lupus erythematosus (SLE)^43^, ^91^ and following infections with malaria^43^, ^92^ and human immunodeficieny virus (HIV).^43^, ^93^ We also showed that plasmablasts were dramatically decreased in the blood of adults and of older children (10–18 years old). Thus, our work here identifies many changes to the immune system, some that complement existing studies and other changes that provide important new information about the composition of the immune system, especially in early life.

Although there is some controversy surrounding whether the womb is sterile or is exposed to microbes from the mother, a critical review of the area does not support the presence of microbiota within the healthy fetal milieu.^60^ Therefore, our analysis focused on comparing the immune system in newborns (i.e. when the immune system is most immature) with all other age groups. We observed significant changes to total B cells, CD56^bright^ NK cells, plasmablasts and double‐negative (DN) T cells within the first 12 months of life. These cells likely play an important role in host defense in the absence of a more developed immune system and the changes we observed in these populations are probably a consequence of pathogen exposure and/or a high vaccine regime in early life.

The immature nature of the immune system in newborns is supported by our data and those of others which show a shift from naïve lymphoid and myeloid cells to a memory phenotype and an accumulation of memory cells with aging.^73^, ^74^, ^87^, ^94^, ^95^, ^96^ In this study, we showed that CD8^+^ T cells experienced the greatest change from naïve to memory cells.^73^, ^74^ This could be a result of thymus atrophy, which reduces thymic output of developing T cells and/or exposure to environmental antigens and pathogens that leads to T‐cell activation and the formation of immunological memory. Our results highlight previously unappreciated changes in infants and children. For example, CD8^+^ T_EM_ and CD4^+^ T_EM_ cells experienced a rise in frequency early in life, 7–12 months and 5–9 years, respectively. Notably, Kang *et al*.^97^ reported a lower proportion of CD4^+^ T_EM_ cells in healthy individuals above 65 years old compared with the adults about 40 years old of age, although we did not observe this difference. CD4^+^ T_CM_ cells were also significantly increased in the 5–9‐year age group, continuing to increase throughout life, while significant increases in CD8^+^ T_CM_ cells were only observed in individuals aged 40 and above. Given that T_CM_ cells express the homing receptors (i.e. CCR7), it is possible that these cells are migrating to lymph nodes, where they are known to provide protection against various pathogens.^47^, ^97^ Regarding the presence of terminally differentiated T cells in peripheral blood, we observed that CD8^+^ T_EMRA_ cells exhibited exponential growth, greatly increasing over time, whereas substantial populations of CD4^+^ T_EMRA_ cells were only detected in individuals aged 40 and above. The accumulation of T_EMRA_ cells in the blood of healthy individuals could be attributed to pathogens such as HCMV which commonly infects humans.^95^, ^98^, ^99^, ^100^ A previous study reported a minor proportion of CD4^+^ T_EMRA_ cells in the blood^101^ and these cells appear elevated in patients infected with Dengue virus.^102^ Among the TH subsets analyzed, TH2 cells were greatly increased over time, while TH1 and TH17 subsets exhibited a bell‐shaped curve, peaking in the 10–18‐year age group and gradually decreasing throughout life. Given that TH2 cells are important for immune responses such as allergy,^52^, ^53^ understanding the mechanisms that underpin the expansion of these cells and the impact of susceptibility to different allergens requires much further investigation. Moreover, because the different subsets of TH cells have profound functional roles, the age‐related changes shown here for these subsets could influence the immune response to various human diseases including infection, autoimmune disorders and cancer.^87^, ^103^, ^104^, ^105^, ^106^, ^107^

Glynn and Moss^1^ revealed that school‐age children were less likely to suffer from severe infectious diseases, suggesting superior immune protection in children. This is interesting as we show that the highest proportions of CD4^+^ T_EM_ cells, memory B cells, plasmablasts, CD11c^+^ B cells and Vδ2^+^ γδ T cells are in children in the 5–9‐year age group, whereas TH1, TH17 and DCs reached their peak in children aged 10–18 years. MAIT cells reached their peak in adults aged 19–30 years; however, the highest proportion of MAIT cells was observed in one child aged between 10 and 18 years (40% of CD3^+^ T cells). Interestingly, we found that MAIT cells were almost undetectable in children up to 4 years of age, before rising in frequency until adulthood and then trending lower in later life, which fits with multiple studies that examined MAIT cell frequency at various timepoints in life.^85^, ^86^ Vδ2^+^ γδ T cells were also low in infants and the elderly, and highest in children of schooling‐age (in the 5–9‐year age group). Given that MAIT cells and Vδ2^+^ γδ T cells can rapidly respond to harmful microorganisms,^59^, ^80^, ^81^, ^106^, ^108^, ^109^ fluctuations in numbers might be detrimental for some diseases, while being beneficial for others. Parrot *et al*.^109^ recently reported a correlation between poor clinical outcome of COVID‐19 and activation of MAIT cells in the blood of patients, while other studies reported a decline in circulating MAIT cells following tuberculosis infection, which correlated with their ability to migrate to tissues to mount an antimicrobial immune response.^109^, ^110^ One important question is whether vaccination programs could be modified to target unconventional T cells to improve host immunity to infectious diseases in susceptible age groups. In line with this suggestion, two recent studies from the same group revealed that MAIT cells and Vδ2^+^ γδ T cells were activated and contributed to the immunogenicity of adenovirus vectors,^111^, ^112^ suggesting that these cells could be used to boost host immunity.

Our data highlight that the immune system is undeveloped or immature in early life, with low proportions of DN T cells, memory T and B cells and of CD16^+^CD56^dim^ and CD56^bright^ NK cells in infants after birth. This may help to explain why infants < 14 months of age are less likely to reject heart transplants, even if the heart is ABO mismatched, because the immune system is still largely immature.^23^, ^24^, ^25^ Interestingly, a recent high‐profile study reported the first pig to human heart transplant in an adult recipient^113^ and although the patient eventually died, it opens up the possibility of using animals as a source of vital organs when human donors are unavailable. Could this be extended to infants where the immune system is immature and less susceptible to rejection, perhaps as a temporary solution until a human organ becomes available?^114^ A greater understanding of how the immune system changes following transplantation is required, but our work here serves as a baseline for changes caused by transplantation.

While our work greatly advances our understanding of the immune system throughout life, further studies would complement our findings. Ontogeny analysis of whole blood would permit the study of granulocytes that are removed during Ficoll‐Paque isolation of PBMCs. Similarly, our antibody cocktails were not broad enough to include the study of innate lymphoid cells, which are the innate counterparts of TH1, TH2 and TH17 cells and may also change throughout life as a consequence of human disease. Moreover, our work did not account for changes to the immune system caused by differences in the metabolic state of an individual (i.e. obese *vs*. lean people). Greater samples sizes would facilitate more detailed investigation into the influence of gender on the composition of the cellular immune system, building on findings we made in this study such as small differences in naïve CD8^+^ T cells, CD8^+^ T_EMRA_ cells, CD4^+^_TEMRA_ cells, transitional B cells and myeloid DCs in males and females aged between 41 and 60 years. Immune phenotyping of immune cells from other human tissues and fluids would also add to our findings from the peripheral blood and provide a more complete picture of the human immune system. For example, MAIT cells are thought to comprise up to 45% of T cells within human adult liver,^80^ thus understanding how these and other immune cells change in tissues would be valuable.

Taken together, we provide an atlas of human immune system in blood and identify major changes to innate and adaptive immune cells throughout life, with comprehensive analysis of infants and children. This work will serve as a basis for understanding changes to the human immune system associated with human diseases and may help to identify immune cell biomarkers that can be exploited as therapeutic or vaccine targets to protect high‐risk groups such as the young and the elderly.

## METHODS

### Ethics Statement

Healthy adult blood samples were obtained from the Australian Red Cross Lifeblood under ethics approval 18‐10VlC‐05, with ethics approval from The Royal Children's Hospital Melbourne Human Research Ethics Committee (HREC38240). Neonatal and pediatric blood samples were taken from consented cardiac surgery patients (HREC38192) and obtained from the Melbourne Children's Heart Tissue Bank for this analysis. Samples were processed by the Murdoch Children's Research Institute Biobank and stored in vapor‐phase liquid nitrogen for less than 18 months.

### Human peripheral blood mononuclear cells

PBMCs from infant, children and adults were isolated using Ficoll‐Paque, frozen in freezing media (10% dimethyl sulfoxide, 90% fetal bovine serum) and stored in liquid nitrogen, prior to thawing and flow cytometric analysis. In total, 36 infants less than 2 years of age, 23 children from 2 to 18 years old and 37 adults from 19 years old to 75 years old were analyzed in this study (Supplementary table 1).

### High‐dimensional flow cytometry analysis

Human PBMCs were stained with Zombie NIR from BioLegend and the surface antibodies listed in Supplementary tables 3 and 4. Antibodies for chemokines were stained at room temperature and all other surface antibodies were stained on ice for 20 min. Cells were analyzed using a 5‐laser Cytek Aurora and the data were processed using FlowJo software (10.8 version; BD, Franklin Lakes, New Jersey, USA). Live human cells were gated on Zombie NIR‐negative cells.

### Data analysis

The data were analyzed using both manual and computational methods in parallel. Manual gating was performed using FlowJo version 10.8, where major immune cell populations were identified based on the gating strategy in Supplementary figure 1. For computational analysis, live intact cells were exported from FlowJo, and analyzed using the Specter R toolkit (version 1.0.0).^61^ Initially, samples were transformed using ArcSinh transformation using a cofactor between 1000 and 5000 depending on the individual channel. Subsequently cells were subject to batch alignment using rPCA from the Seurat toolkit (version 4.0.5),^62^ implemented in Specter. In brief, rPCA projects cells from one data set into the PCA space of another, where cells that share a common state across the data sets are then anchored together using mutual nearest neighbors. These anchor pairs allow for the correction of expression values across the data set, effectively unifying the data sets into a single analysis, mitigating the influence of technical sources of variation while preserving biologically relevant differences between cells. In our analysis, each sample was integrated independently with a chosen reference sample from the 0–1‐month age group. Following integration, cells were clustered using FlowSOM (version 2.0.0),^115^ visualized using fast Fourier transform–accelerated interpolation‐based T‐distributed stochastic neighbor embedding (FIt‐SNE) (version 1.2.1)^116^ and annotated at the lineage and broad population level. Each lineage was then isolated and subject to subclustering and detailed annotation, before unifying the lineages to create a comprehensive map of immune states over time. The statistical analysis was performed using the Kruskal–Wallis nonparametric test with Dunn's multiple comparisons test, comparing the 0–1‐month age group with all other age groups through GraphPad Prism version 9.2.0 software (GraphPad Software Inc, San Diego, CA, USA) and *P*‐value < 0.05 was considered significant.

## AUTHOR CONTRIBUTIONS

SJ, CMH, MA and TMA performed methodology, formal analysis, data curation, visualization, and validation data. SJ, IEK, TMA and DGP were involved in conceptualization of the study, writing the original draft and writing, reviewing and editing all aspects of the manuscript. ATP, CMH, SL, JA, PVL, SPB, MA, KA, and LP contributed to project administration, resources and reviewing and editing the manuscript. IEK, PVL and DGP were involved in supervision and funding acquisition.

## CONFLICT OF INTEREST

The authors declare no conflicts of interest.

## Data Availability

Most data generated in this study are included in this published article. Additional data are available from the corresponding authors upon reasonable request. Source data are provided with this paper and a link for this work will be provided within the publication.
