# Supplementary material for: A high‐dimensional cytometry atlas of peripheral blood over the human life span
Source: Immunol Cell Biol. 2022 Nov 6;100(10):805–21. doi: 10.1111/imcb.12594 (PMC9828744; doi:10.1111/imcb.12594)
Supplement: Supplementary file 1 [file IMCB-100-805-s001.pdf]

Supplementary table 1.

**Groups**	**Ages range**	**No. of samples**
Group-1	0-1 month	N= 13
Group-2	2-6 months	N= 10
Group-3	7-12 months	N= 7
Group-4	13-24 months	N= 6
Group-5	3-4 years	N= 8
Group-6	5-9 years	N= 7
Group-7	10-18 years	N= 8
Group-8	19-30 years	N= 7
Group-9	31-40 years	N= 6
Group-10	41-60 years	N= 12
Group-11	61-75 years	N= 12

Supplementary table 2. Immune cells markers of peripheral blood mononuclear cell (PBMCs).

**Cell populations**	**Markers**
$CD3^+$ T cells	$ZombieNIR^-$ $CD3^+$
$CD4^+$ T cells	$CD3^+$ $CD4^+$ $CD8^-$
$CD8^+$ T cells	$CD3^+$ $CD8^+$ $CD4^-$
Double negative T cells	$CD3^+$ $CD4^-$ $CD8^-$
Naïve $CD4^+$ T cells	$CD3^+$ $CD4^+$ $CCR7^+$ $CD45RA^+$
TCM $CD4^+$ T cells	$CD3^+$ $CD4^+$ $CCR7^+$ $CD45RA^-$
TEM $CD4^+$ T cells	$CD3^+$ $CD4^+$ $CCR7^-$ $CD45RA^-$
TEMRA $CD4^+$ T cells	$CD3^+$ $CD4^+$ $CCR7^-$ $CD45RA^+$
Naïve $CD8^+$ T cells	$CD3^+$ $CD8^+$ $CCR7^+$ $CD45RA^+$
TCM $CD8^+$ T cells	$CD3^+$ $CD8^+$ $CCR7^+$ $CD45RA^-$
TEM $CD8^+$ T cells	$CD3^+$ $CD8^+$ $CCR7^-$ $CD45RA^-$
TEMRA $CD8^+$ T cells	$CD3^+$ $CD8^+$ $CCR7^-$ $CD45RA^+$
Regulatory T cells (Treg)	$CD3^+$ $CD4^+$ $CD25^+$ $CD127^-$
Naïve Treg	$CD3^+$ $CD4^+$ $CD25^+$ $CD127^-$ $CD45RA^+$
Memory Treg	$CD3^+$ $CD4^+$ $CD25^+$ $CD127^-$ $CD45RA^-$
T helper (TH)1 cells	$CD3^+$ $CD4^+$ $CXCR3^+$ $CCR6^-$
TH2 cells	$CD3^+$ $CD4^+$ $CXCR3^-$ $CCR4^+$ $CCR6^-$
TH17 cells	$CD3^+$ $CD4^+$ $CXCR3^-$ $CCR4^+$ $CCR6^+$ $CD161^+$
$Vd2^+$ $\gamma\delta$ T cells	$CD3^+$ $TCR\gamma\delta^+$ $V\delta2^+$
$Vd2^-$ $\gamma\delta$ T cells	$CD3^+$ $TCR\gamma\delta^+$ $V\delta2^-$
MAIT cells	$CD3^+$ $CD161^+$ $V\alpha7.2^+$
B cells	$CD3^-$ $CD19^+$ $CD20^+$
IgD^+CD27^+ B cells	$CD3^-$ $CD19^+$ $CD20^+$ IgD^+ $CD27^+$
IgD^+CD27^- B cells	$CD3^-$ $CD19^+$ $CD20^+$ IgD^+ $CD27^-$
IgD^-CD27^+ B cells	$CD3^-$ $CD19^+$ $CD20^+$ IgD^- $CD27^+$
IgD^-CD27^- B cells	$CD3^-$ $CD19^+$ $CD20^+$ IgD^- $CD27^-$
$CD11c^+$ B cells	$CD3^-$ $CD19^+$ $CD20^+$ $CD11c^+$
Transitional B cells	$CD3^-$ $CD19^+$ $CD24^+$ $CD38^+$

Plasmablasts	$CD3^- CD19^+ CD20^- CD38^+$
$CD56^{dim}$ NK cells	$CD3^- CD19^- CD20^- CD14^- CD56^{dim}$
$CD56^{bright}$ NK cells	$CD3^- CD19^- CD20^- CD14^- CD56^{bright}$
$CD16^+CD57^+$ $CD56^{dim}$ NK cells	$CD3^- CD19^- CD20^- CD14^- CD56^{dim} CD16^+ CD57^+$
$CD16^+CD57^-$ $CD56^{dim}$ NK cells	$CD3^- CD19^- CD20^- CD14^- CD56^{dim} CD16^+ CD57^-$
$CD16^-CD57^+$ $CD56^{dim}$ NK cells	$CD3^- CD19^- CD20^- CD14^- CD56^{dim} CD16^- CD57^+$
$CD16^-CD57^-$ $CD56^{dim}$ NK cells	$CD3^- CD19^- CD20^- CD14^- CD56^{dim} CD16^- CD57^-$
$CD16^+CD57^+$ $CD56^{bright}$ NK cells	$CD3^- CD19^- CD20^- CD14^- CD56^{bright} CD16^+ CD57^+$
$CD16^+CD57^-$ $CD56^{bright}$ NK cells	$CD3^- CD19^- CD20^- CD14^- CD56^{bright} CD16^+ CD57^-$
$CD16^-CD57^+$ $CD56^{bright}$ NK cells	$CD3^- CD19^- CD20^- CD14^- CD56^{bright} CD16^- CD57^+$
$CD16^-CD57^-$ $CD56^{bright}$ NK cells	$CD3^- CD19^- CD20^- CD14^- CD56^{bright} CD16^- CD57^-$
$NKG2A^+NKG2C^+$ $CD56^{dim}$ NK cells	$CD3^- CD19^- CD20^- CD14^- CD56^{dim} NKG2A^+ NKG2C^+$
$NKG2A^+NKG2C^-$ $CD56^{dim}$ NK cells	$CD3^- CD19^- CD20^- CD14^- CD56^{dim} NKG2A^+ NKG2C^-$
$NKG2A^-NKG2C^+$ $CD56^{dim}$ NK cells	$CD3^- CD19^- CD20^- CD14^- CD56^{dim} NKG2A^- NKG2C^+$
$NKG2A^-NKG2C^-$ $CD56^{dim}$ NK cells	$CD3^- CD19^- CD20^- CD14^- CD56^{dim} NKG2A^- NKG2C^-$
$NKG2A^+NKG2C^+$ $CD56^{bright}$ NK cells	$CD3^- CD19^- CD20^- CD14^- CD56^{bright} NKG2A^+ NKG2C^+$
$NKG2A^+NKG2C^-$ $CD56^{bright}$ NK cells	$CD3^- CD19^- CD20^- CD14^- CD56^{bright} NKG2A^+ NKG2C^-$
$NKG2A^-NKG2C^+$ $CD56^{bright}$ NK cells	$CD3^- CD19^- CD20^- CD14^- CD56^{bright} NKG2A^- NKG2C^+$
$NKG2A^-NKG2C^-$ $CD56^{bright}$ NK cells	$CD3^- CD19^- CD20^- CD14^- CD56^{bright} NKG2A^- NKG2C^-$
Classical monocytes	$CD3^- CD19^- CD20^- CD56^- CD14^+ CD16^-$
Intermediate monocytes	$CD3^- CD19^- CD20^- CD56^- CD14^+ CD16^+$
Non-classical monocytes	$CD3^- CD19^- CD20^- CD56^- CD14^- CD16^+$
Dendritic cells (DCs)	$CD3^- CD19^- CD20^- CD56^- CD14^- CD16^-$
Myeloid dendritic cells (mDCs)	$CD3^- CD19^- CD20^- CD56^- CD14^- CD16^- CD11c^+$
Plasmacytoid dendritic cells (pDCs)	$CD3^- CD19^- CD20^- CD56^- CD14^- CD16^- CD11c^- CD123^+$

Supplementary table 3. Antibody cocktails to define different subsets of T cells of PBMCs.

Antibody	Colour	Manufacturer	Clone
CD197 (CCR7)	BV785	BioLegend, San Diego, USA	G043H7
CD183 (CXCR3)	APC	BD Biosciences, San Diego, CA, USA	1C6
CD196 (CCR6)	BUV496	BD Biosciences, San Diego, CA, USA	11A9
CD194 (CCR4)	BV605	BioLegend, San Diego, USA	L291H4
TCRγδ	FITC	BD Biosciences, San Diego, CA, USA	11F2
CD127	APC-R700	BD Biosciences, San Diego, CA, USA	HIL-7R-M21
CD45RA	PerCP/cy5.5	BD Biosciences, San Diego, CA, USA	HI100
CD25	PE-CF594	BD Biosciences, San Diego, CA, USA	M-A251
Vδ2 TCR	PE	BioLegend, San Diego, USA	B6
CD161	PEvio770	Miltenyi Biotec, New South Wales, Australia	191B8
CD3	BUV395	BD Biosciences, San Diego, CA, USA	UCHT1
CD27	BUV737	BD Biosciences, San Diego, CA, USA	L128
Vα7.2 TCR	BV711	BioLegend, San Diego, USA	3C10
CD4	BV421	BD Biosciences, San Diego, CA, USA	RPA-T4
CD8a	BUV805	BD Biosciences, San Diego, CA, USA	SK1
CD56	BV510	BioLegend, San Diego, USA	HCD56
Zombie NIR	NIR	BioLegend, San Diego, USA	

Supplementary table 4. Antibody cocktails to define different subsets of B cells and innate cells of PBMCs.

Antibody	Colour	Manufacturer	Clone
CD3	PerCP/cy5.5	BD Biosciences, San Diego, CA, USA	UCHT1
CD24	BV711	BioLegend, San Diego, USA	ML5
CD16	BV605	BD Biosciences, San Diego, CA, USA	3G8
HLA-DR	APC	BD Biosciences, San Diego, CA, USA	G46-6
CD11c	FITC	BioLegend, San Diego, USA	Bu15
CD20	BV421	BioLegend, San Diego, USA	2H7
CD56	BV510	BioLegend, San Diego, USA	HCD56
CD159c (NKG2C)	AF700	R&D Systems, Minneapolis, USA	#134591
CD159a (NKG2A)	PE	Beckman Coulter, New South Wales, Australia	Z199
CD14	BUV805	BD Biosciences, San Diego, CA, USA	M5E2
CD123	PECy7	BD Biosciences, San Diego, CA, USA	7G3
CD27	BUV737	BD Biosciences, San Diego, CA, USA	L128
IgD	BUV395	BD Biosciences, San Diego, CA, USA	IA6-2
CD19	BV785	BioLegend, San Diego, USA	HIB19
CD86	BV650	BD Biosciences, San Diego, CA, USA	2331 (FUN-1)
CD38	BUV496	BD Biosciences, San Diego, CA, USA	HIT2
CD57	PE-CF594	BD Biosciences, San Diego, CA, USA	NK-1
Zombie NIR	NIR	BioLegend, San Diego, USA	

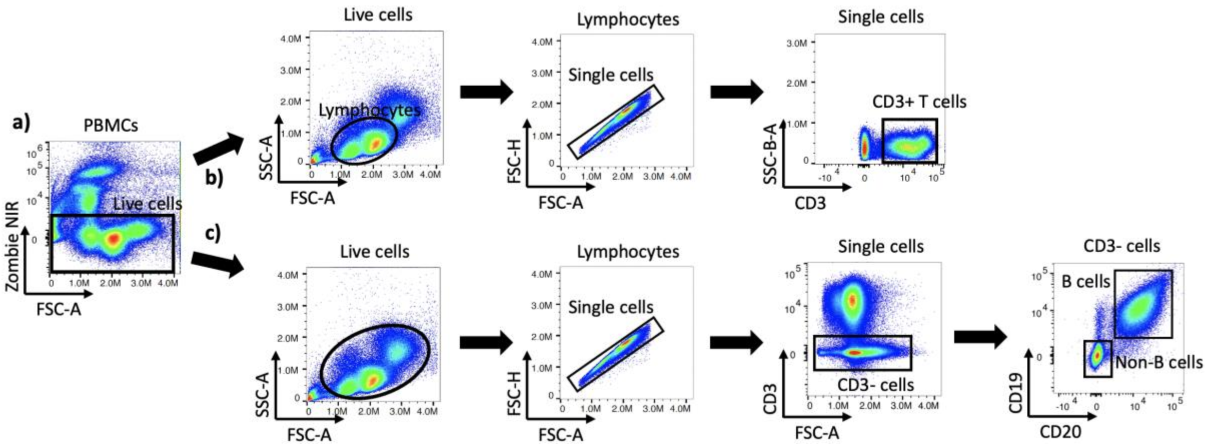

Supplementary figure 1. Gating strategy to identify CD3$^+$ T cells and CD3$^-$ cells. Representative flowcytometry plots from PBMCs of one of the study participants show the gating strategy identifying CD3$^+$ T cells and CD3$^-$ cells. **a)** Live cells were gated with FSC-A vs ZombieNIR. **b)** Lymphocytes were gated on FSC-A vs SSC-A and singlet cells were gated on FSC-A vs FSC-H. From live single cells, CD3$^+$ T cells were gated on CD3 vs SSC-B-A. **c)** Lymphocytes and large granulocytes were gated on FSC-A vs SSC-A and singlet cells were gated on FSC-A vs FSC-H. Next, CD3$^-$ cells were gated on CD3 vs FSC-A. From CD3$^-$ cells, B cells and non-B cells were gated on CD20 vs CD19.

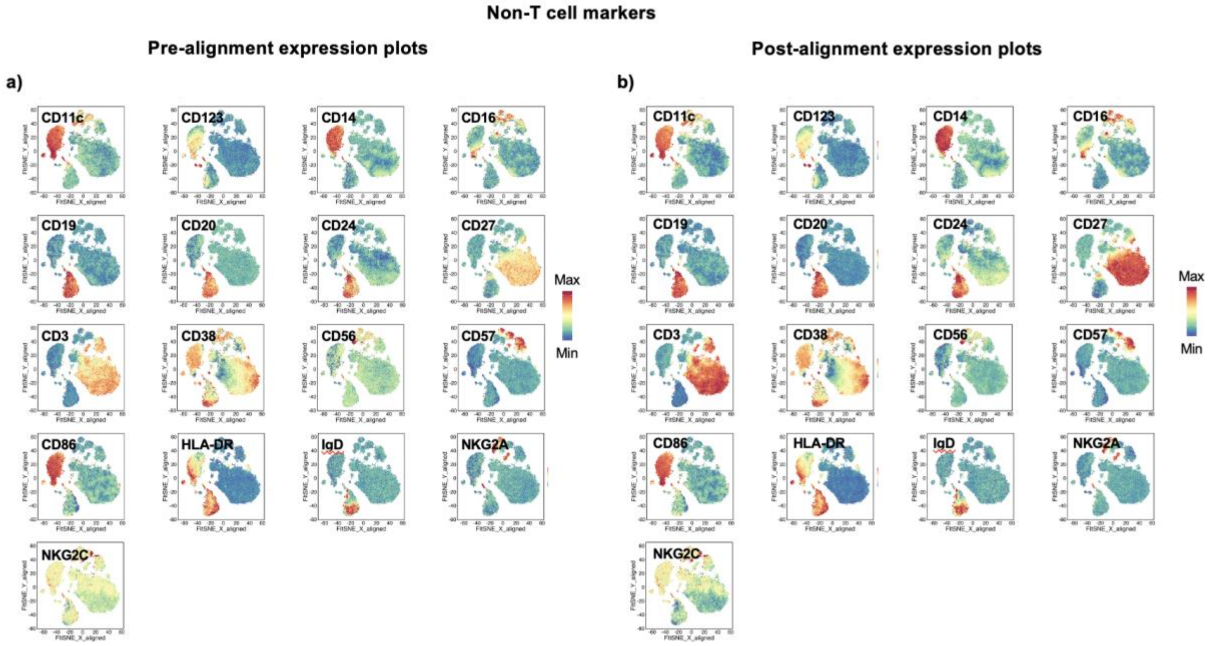

Supplementary figure 2. Non-T cell panel batch alignment. FIt-SNE plots generated using aligned markers. Each marker is displayed on the coloured FIt-SNE plot, where the colour indicates the level of expression of either pre-alignment **(a)** or post-alignment **(b)** values.

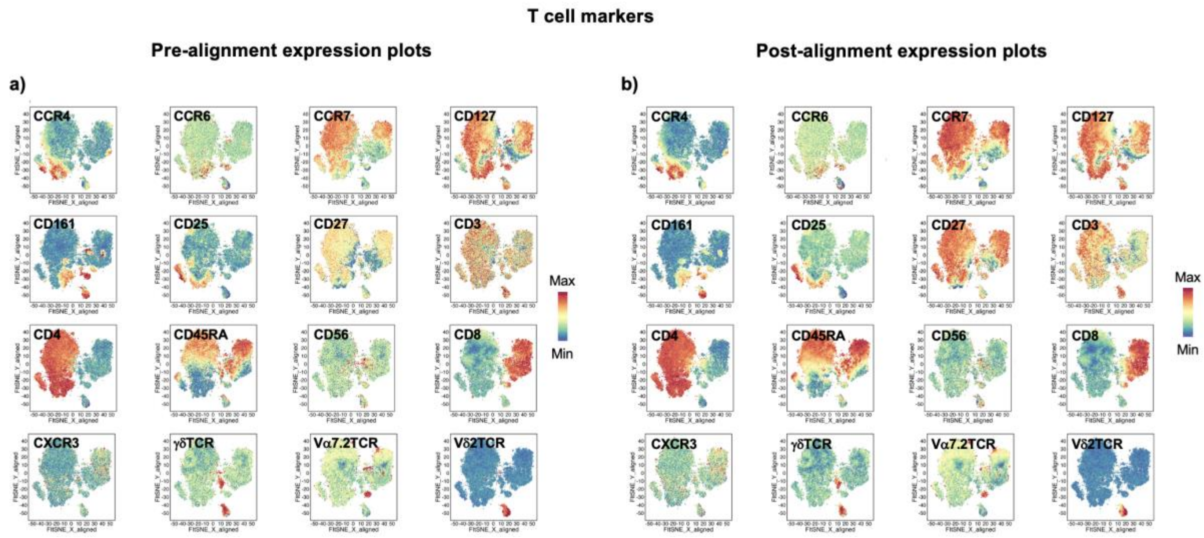

Supplementary figure 3. T cell panel batch alignment. FIt-SNE plots generated using aligned markers. Each marker is displayed on the coloured FIt-SNE plot, where the colour indicates the level of expression of either pre-alignment **(a)** or post-alignment **(b)** values.

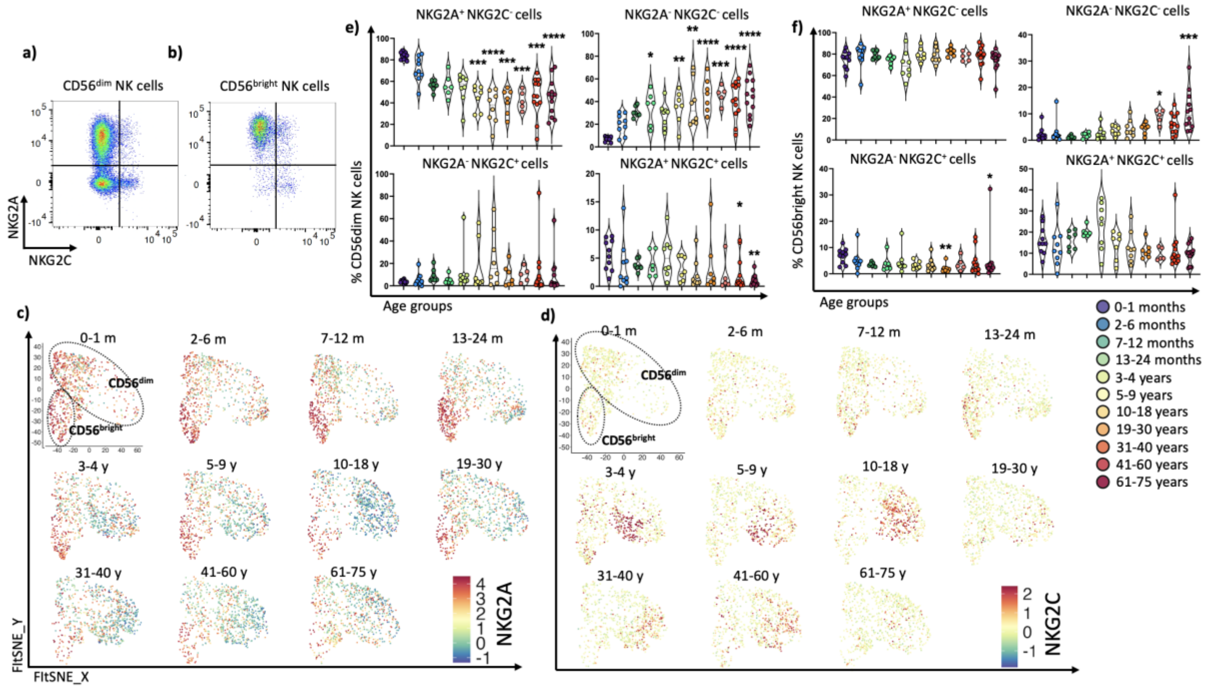

Supplementary figure 4. The proportion of NKG2A and NKG2C receptors in different subsets of NK cells is varied and aging significantly affects them. CD56^{dim} NK cells and CD56^{bright} NK cells of PBMCs from 96 individuals were used for flow cytometric analysis. **a-b)** Representative two-parameter density flowcytometry plots of PBMCs from one of the study participants show different subsets of CD56^{dim} and CD56^{bright} NK cells based on the expression of NKG2A vs NKG2C. **c-d)** The expression of NKG2A and NKG2C in the eleven age groups was validated with unsupervised FIt-SNE analysis using a blue-red continues colour scale. **e-f)** Individual-value violin plots show the proportion of NKG2A^{+}NKG2C^{+}, NKG2A^{+}NKG2C^{-}, NKG2A^{-}NKG2C^{+}, and NKG2A^{-}NKG2C^{-} of both CD56^{dim} NK cells **(e)** and CD56^{bright} NK cells **(f)** among the eleven age groups. Data is shown with median. Each dot represents one participant, and each colour represents one age group. The non-parametric Kruskal-Wallis test with Dunn's multiple comparisons test was used comparing 0-1 m age group to all other age groups. *P*-values are $P > 0.05$ (ns), $P \leq 0.05$ (*), $P \leq 0.01$ (**), $P \leq 0.001$ (***), $P \leq 0.0001$ (****).

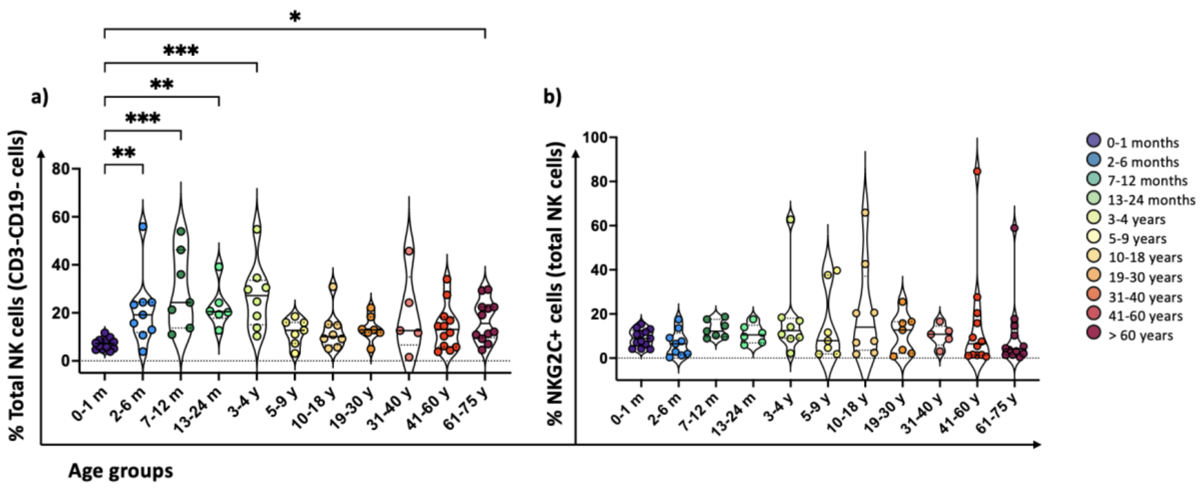

Supplementary figure 5. The proportion of total NK cells and NKG2C⁺ NK cells from 96 individuals were used for flow cytometric analysis. **a, b)** Individual-value violin plots show the proportion of total NK cells **(a)** and NKG2C⁺ NK cells **(b)** among the eleven age groups. Data is shown with median. Each dot represents one participant, and each colour represents one age group. The non-parametric Kruskal-Wallis test with Dunn's multiple comparisons test was used comparing 0-1 m age group to all other age groups. *P*-values are $P > 0.05$ (ns), $P \leq 0.05$ (*), $P \leq 0.01$ (**), $P \leq 0.001$ (***), $P \leq 0.0001$ (****).

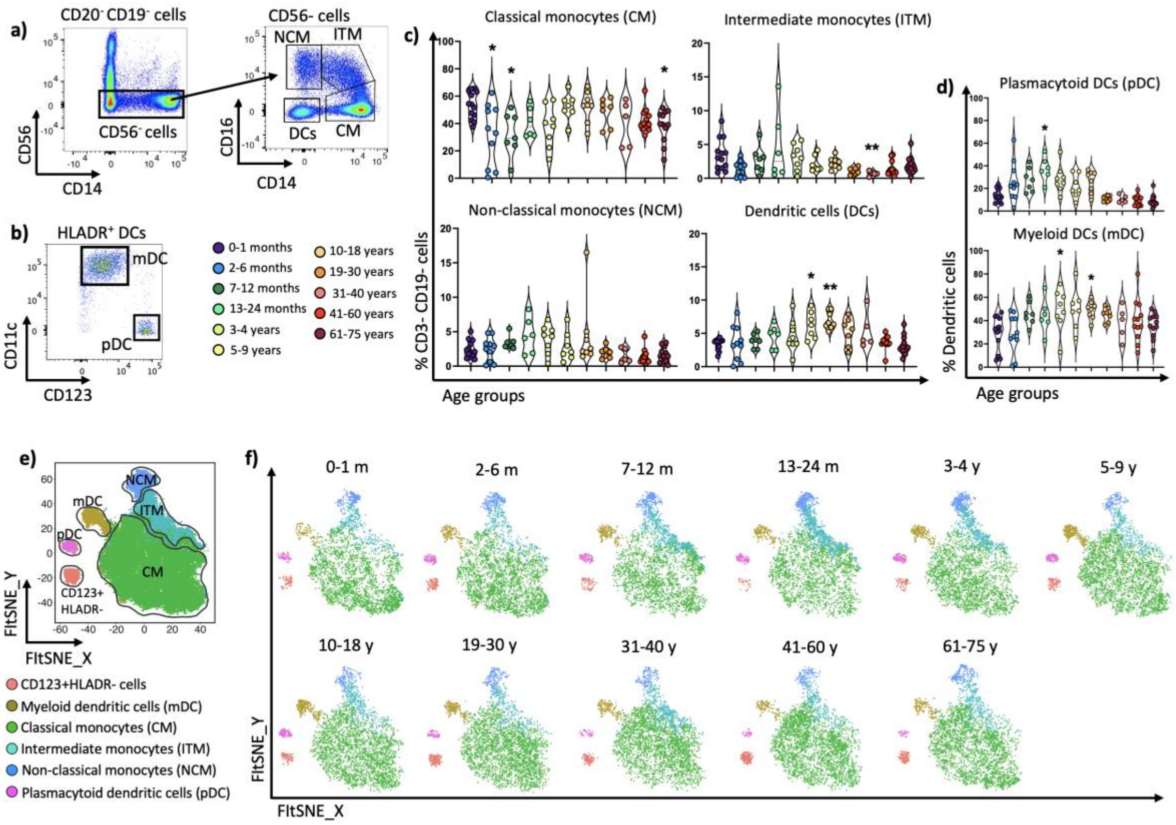

Supplementary figure 6. The proportion of monocyte subsets and dendritic cell subsets were higher in children compared with adults. Monocytes and dendritic cells of PBMCs from 96 individuals were used for flow cytometric analysis. **a)** Representative two-parameter density flowcytometry plots of PBMCs from one of the study participants show classical (CM), non-classical (NCM), intermediate (ITM) monocytes, total dendritic cells (DCs), **b)** myeloid DCs (mDC) and plasmacytoid DCs (pDC). **c-d)** Individual-value violin plots represent the proportion of CM, NCM, ITM, DCs and mDC and pDC cells among the eleven age groups. Data is shown with median. Each dot represents one participant, and each colour represents one age group. **e-f)** The transition of different monocytes and dendritic cell subsets in the eleven age groups was validated with unsupervised FIt-SNE analysis. Each colour represents a cell subset. The non-parametric Kruskal-Wallis test with Dunn's multiple comparisons test was used comparing 0-1 m age group to all other age groups. *P*-values are $P > 0.05$ (ns), $P \leq 0.05$ (*), $P \leq 0.01$ (**), $P \leq 0.001$ (***), $P \leq 0.0001$ (****).

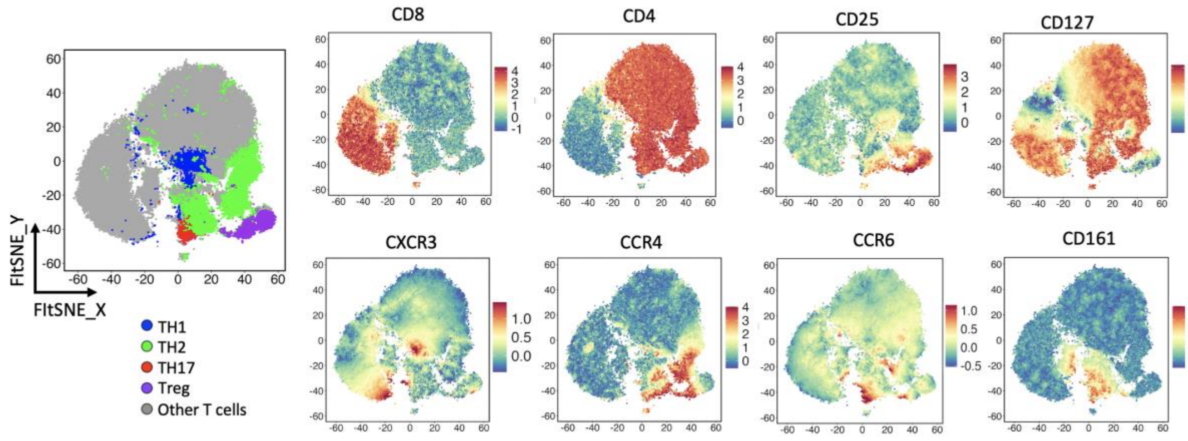

Supplementary figure 7. Ninety-six PBMC samples were merged to create a single FIt-SNE map of eight phenotypic markers: CD8, CD4, CD25, CD127, CXCR3, CCR4, CCR6, CD161 defining different subsets of helper T (TH) cells and regulatory T (T_{reg}) cells using a blue-red continuous colour scale. FIt-SNE analysis was performed using 750 iterations with a perplexity of 200.

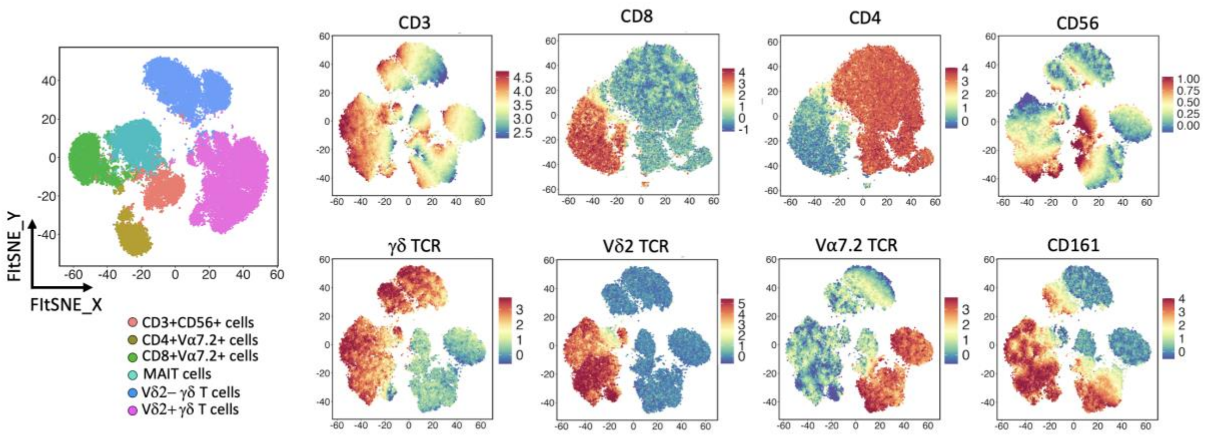

Supplementary figure 8. Ninety-six PBMC samples were merged to create a single FIt-SNE map of eight phenotypic markers: CD3, CD4, CD8, CD56, γδTCR, Vδ2 TCR, Vα7.2 TCR, CD161 defining different subsets of unconventional T cells using a blue-green-red continuous colour scale. FIt-SNE analysis was performed using 750 iterations with a perplexity of 200.

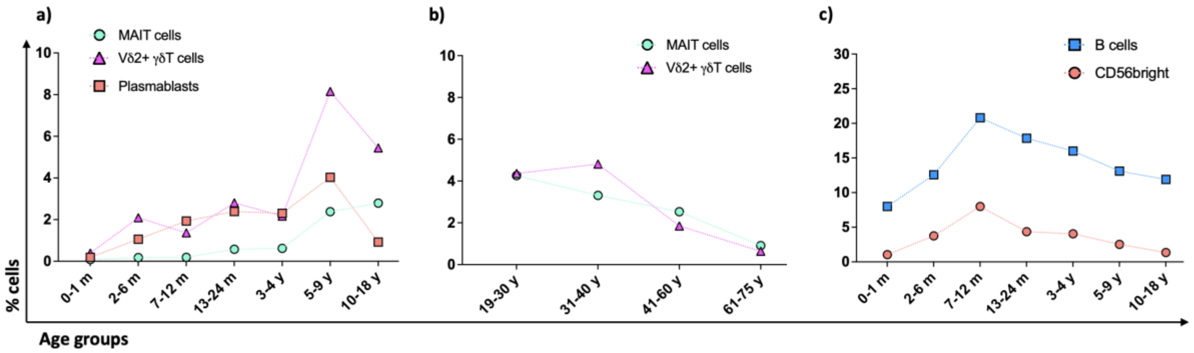

Supplementary figure 9. Correlation studies of immune cell subsets throughout ontogeny. The correlation between the proportion of plasmablasts (orange) from innate immunity and unconventional T cells such as MAIT cells (turquoise) and Vδ2$^+$ $\gamma\delta$ T cells (pink) have been shown in children **(A)** and adults **(B)**. Moreover, the correlation between the proportion of total B cells (blue) and CD56^{bright} NK cells (orange) in different age groups of children **(C)** have been shown. Data is shown with median of each age group.

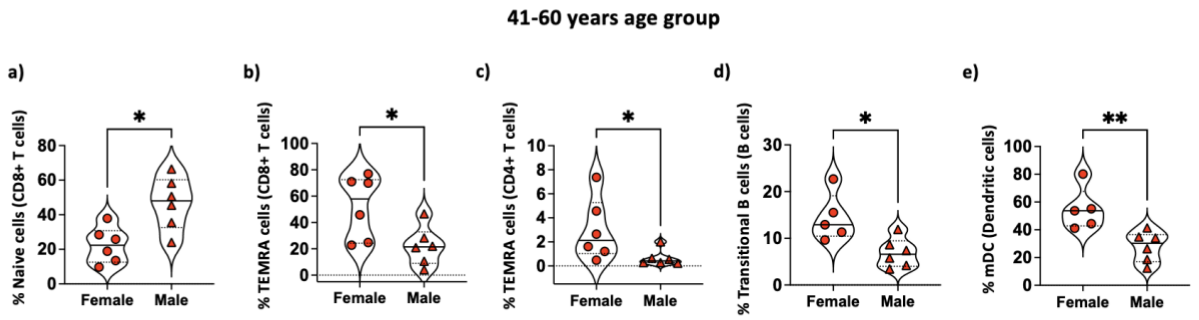

Supplementary figure 10. Gender affects the proportion of some cell subsets in peripheral blood of people in 41-60 years old age group. Proportion of CD8^{+} naive cells **(a)**, CD8^{+} $_{TEMRA}$ cells **(b)**, CD4^{+} T$_{EMRA}$ cells **(c)**, transitional B cells **(d)**, and myeloid DCs (mDC) **(e)** were compared between females (circle) and males (triangle). The Mann Whitney *U*-test used to compare the frequency of cells between females and males. Data is shown with median. Each dot represents an individual.
